# Recent Tumor Necrosis Factor-Related Apoptosis-Inducing Ligand Engineering Strategies for Precise Strike Therapy against Tumor

**DOI:** 10.34133/bmr.0170

**Published:** 2025-03-19

**Authors:** Chae Eun Lee, Kyung Mu Noh, Sungjun Kim, Jiyeon Hong, Kyobum Kim

**Affiliations:** ^1^ Department of Chemical and Biochemical Engineering, Dongguk University, Seoul 04620, Republic of Korea.; ^2^ School of Chemical and Biological Engineering, Institute of Chemical Processes, Seoul National University, Seoul 08826, Republic of Korea.

## Abstract

Effective drug delivery relies on the selection of suitable carriers, which is crucial for protein-based therapeutics such as tumor necrosis factor-related apoptosis-inducing ligand (TRAIL). One of the key advantages of TRAIL is its ability to selectively induce apoptosis in cancer cells excluding healthy tissues by binding to death receptors DR4 and DR5, which are highly expressed in various cancer cells. Despite this promise, the clinical application of TRAIL has been limited by its short half-life, limited stability, and inefficient delivery to tumor sites. To overcome currently available clinical and engineering approaches, a series of sophisticated strategies is required: (a) the design of biomaterial-mediated carriers for enhanced targeting efficacy, particularly via optimizing selected materials, composition, formulation, and surface modulation. Moreover, (b) development of genetically modified cellular products for augmented TRAIL secretion toward tumor microenvironments and (c) cell surface engineering techniques for TRAIL immobilization onto infusible cell populations are also discussed in the present review. Among these approaches, living cell-based carriers offer the distinct advantage of systemically administered TRAIL-functionalized cells capturing circulating tumor cells in the bloodstream, thereby preventing secondary tumor formation. This review provides insight into the development of novel TRAIL delivery platforms, discusses considerations for clinical translation, and suggests future directions and complementary strategies to advance the field of TRAIL-based cancer therapeutics.

## Introduction

Cancer remains one of the most complex and prevalent health challenges worldwide, recognized by its resistance to conventional therapies. Recent cancer treatments encompass a variety of modalities, including surgery, chemotherapy, targeted therapy, radiation, and immunotherapy [1]. Among these, conventional chemotherapy is widely used due to its accessibility, cost-effectiveness, and broad applications. However, a major limitation of chemotherapies is their lack of selectivity for cancer cells, often resulting in substantial off-target effects and damage to healthy tissues [2]. Additionally, chemotherapy induces toxicity to multiple organ systems, including the cardiac, neurological, renal, pulmonary, and hepatic systems in the late stage [3]. Another major challenge of chemotherapy is its dependence on the intrinsic apoptosis pathway, which requires activation of the p53 gene, a crucial regulator of the intrinsic apoptosis pathway. However, mutations in the p53 gene are present in over 50% of human tumors, impairing apoptosis activation and leading to treatment resistance. As a result, alternative therapeutic strategies that circumvent p53 dependency are critically needed to improve cancer treatment outcomes [4].

Tumor necrosis factor (TNF)-related apoptosis-inducing ligand (TRAIL) and Fas ligand (FasL), both members of the TNF superfamily, are crucial proteins that could be involved in inducing extrinsic apoptosis in cancer cells regardless of p53 mutations. These proteins are widely expressed on immune cell surfaces, where their membrane-bound forms interact with death receptors on target cells to initiate apoptotic signaling [5,6]. Due to high systemic toxicity associated with FasL, further research has concentrated on developing TRAIL as a safer alternative for cancer treatment [7]. TRAIL selectively induces extrinsic apoptosis by binding to specific death receptors (DR4 and DR5), which are predominantly expressed on tumor cell surfaces. In contrast, normal cells harbor decoy receptors (DcR1 or DcR2) that suppress apoptotic signaling, thereby evading the off-target effects of TRAIL [8]. Upon binding of TRAIL to DR4 and DR5 on the cancer cell surface, it facilitates the recruitment of the intracellular death-inducing signaling complex (DISC) [9]. This complex forms through the interaction of the Fas-associated death domain adaptor protein, which subsequently recruits caspase-8. Activation of caspase-8 at the DISC initiates a caspase cascade, ultimately guiding the cell along the apoptotic pathway [10]. Given its tumor-selective and p53-independent apoptotic mechanism, TRAIL represents a promising alternative to conventional chemotherapy. Furthermore, clinical trials have demonstrated that TRAIL-based therapies are both safe and well-tolerated, highlighting their potential to improve cancer treatment outcomes [11–13].

TRAIL initiates apoptosis by specifically binding to death receptors DR4 and DR5, highly expressed on many cancer cells yet minimally present on normal cells. Despite the selective apoptosis-inducing properties of TRAIL mediated by DR4/DR5 for cancer cells, clinical trials are constrained by several factors, including its short half-life, poor stability, and challenges in effective delivery to tumor sites [14,15]. The short half-life and instability of TRAIL in the bloodstream reduce its therapeutic effect, complicating the maintenance of therapeutic threshold concentrations over extended periods. For instance, recombinant human TRAIL (rhTRAIL) has a half-life of less than 1 h after administration in human patients with solid tumors, necessitating frequent administrations or higher doses, which can lead to off-target effects and toxicity [12]. Moreover, targeted delivery of TRAIL to tumor sites presents important challenges. TRAIL can navigate the complex and often hostile tumor microenvironment (TME), characterized by abnormal vasculature, high interstitial pressure, and dense extracellular matrices. These architectural and physiological barriers frequently impede the effective penetration and retention of TRAIL in tumor tissue [16]. For example, targeted delivery of TRAIL to tumor sites failed due to rapid systemic clearance and physical barriers in the TME, which reduced the therapeutic dose level of available TRAIL to induce sufficient apoptosis in cancer cells [17,18].

To address these limitations, numerous studies have investigated various engineering strategies to improve TRAIL delivery and efficacy. Exogenous TRAIL delivery strategies aim to enhance pharmacokinetics, targeting specificity, and overall therapeutic impact of TRAIL-based anticancer treatments. These strategies involve advanced biomaterial-mediated drug delivery systems including nanoparticles (NPs), gels, and cell carriers, which are designed to overcome structural and physiological barriers of the TME and achieve precise tumor localization [19,20]. Among the most thoroughly investigated methods is the use of NPs and cells as carriers for TRAIL. NPs can be engineered to display TRAIL externally and deliver it directly to tumor cells, thereby sequentially enhancing its antitumor activity (i.e., apoptosis). Various types of NPs, such as liposomes, polymeric NPs, and iron oxide NPs, have been utilized for TRAIL delivery [15,19]. These carriers can also be functionalized with targeting ligands to ensure specific binding to tumor cells, thus improving therapeutic efficacy. Furthermore, immune cells are promising candidates as TRAIL carriers due to their inherent cancer-targeting properties [20–22]. Functionalizing NPs and transplantable cell surfaces with TRAIL and additional targeting moieties could enhance specific targeting affinity to cancer cell surface receptors. Consequently, this could increase in vivo selectivity of TRAIL for cancer cells, lead to reduced off-target effects, and enhance antitumor activity [20,23,24].

**Fig. 1.**
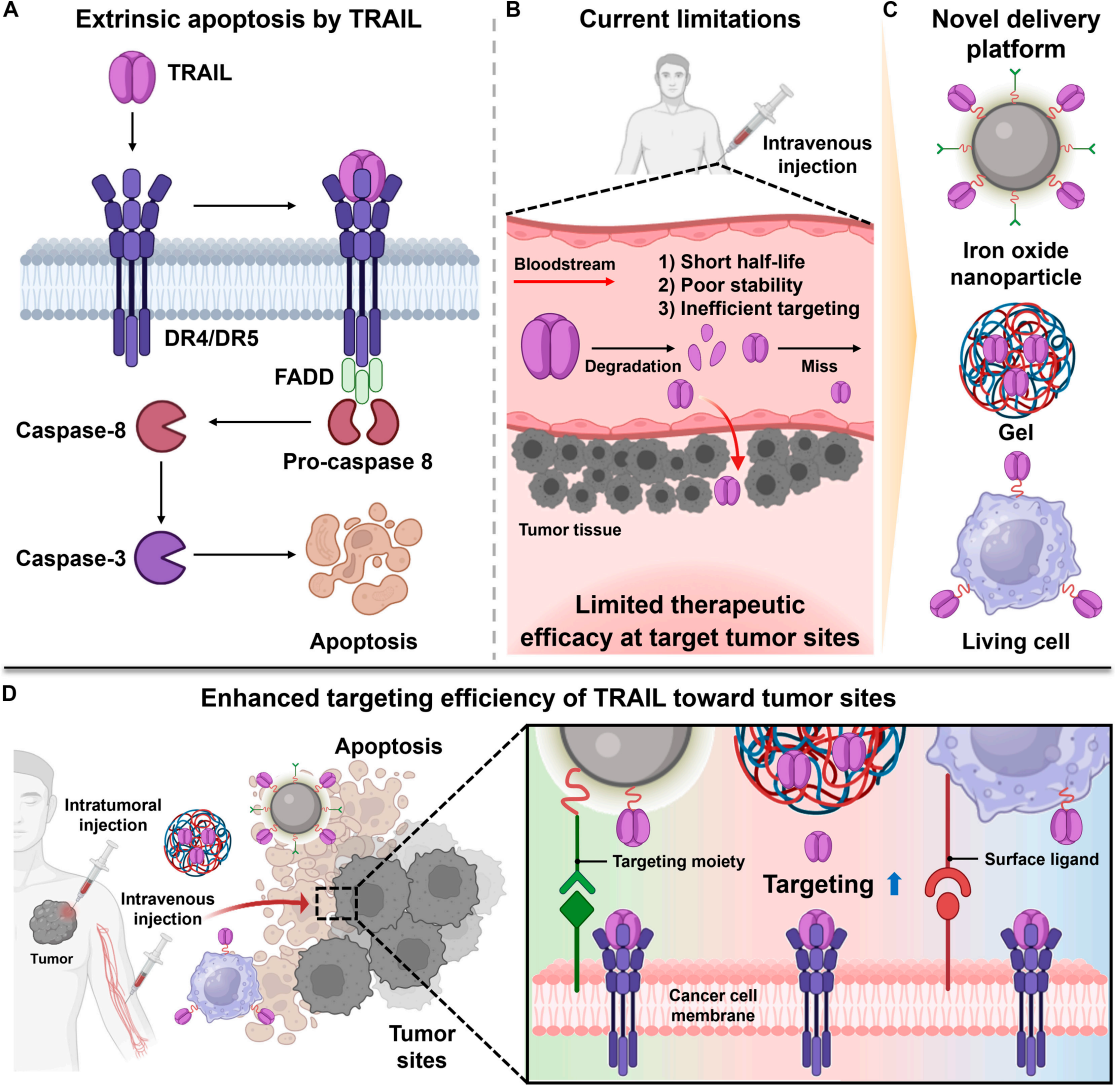
Schematic illustration of current TRAIL-mediated cancer therapies and novel strategies designed to enhance the therapeutic efficacy of TRAIL-based treatment. (A) Apoptosis pathway in cancer cells induced by TRAIL binding. (B) Limitations of TRAIL delivery to tumor sites. (C) Novel TRAIL delivery strategies, including iron oxide, gel, and living cell systems. (D) Improved therapeutic efficacy achieved through advanced TRAIL delivery platforms.

This review offers a comprehensive overview of current strategies for exogenous TRAIL delivery, emphasizing cell-based carriers while addressing ongoing challenges and future perspectives for each approach. We introduce recent engineering techniques in the design and fabrication of NPs and modification of cell carriers to overcome the current limitations of TRAIL-based anticancer treatments. Specifically, we summarized (a) the current status and limitations of TRAIL-based therapies in clinical trials, (b) the therapeutic efficacy of TRAIL-conjugated NPs and encapsulated TRAIL formulations, and (c) innovative cell surface engineering approaches for TRAIL delivery (Fig. 1). By leveraging the unique functionality of TRAIL for selective apoptosis and its established clinical safety, novel TRAIL delivery technologies could lead to the development of more effective anticancer drugs that surpass existing chemotherapy.

## Current Status of TRAIL Therapy in Clinical Trials

Since TRAIL selectively induces apoptosis in cancer cells while sparing normal cells, various formulations of recombinant TRAIL protein and DR4/DR5 binding agonistic antibodies have been evaluated in preclinical models and clinical trials [25,26]. Owing to its mechanism of selectively inducing apoptosis via DR4/DR5 on the surface of target cells, rhTRAIL and DR4/DR5 agonistic antibodies have been developed, demonstrating their safety and tolerability in various clinical trials for cancer treatment (Table S1). rhTRAIL preferentially induces apoptosis in cancer cells and exhibits minimal toxicity when systemically administered to animals. This has led to multiple clinical trials involving biological agents targeting TRAIL receptors, including agonistic antibodies for DR4 and DR5 [27]. These clinical trials have shown that rhTRAIL and its agonistic antibodies selectively kill cancer cells in various solid tumors [12,28]. However, despite therapeutic outcomes in preclinical results and demonstrated safety in clinical trials, TRAIL-based therapies face several limitations. Dulanermin, an rhTRAIL variant, exhibited safety and good tolerability in Phase I trials, with only a limited number of patients showing partial response (PR) or complete responses (CRs) when treated alone or in combination with chemotherapy or rituximab (i.e., CD20-targeting human monoclonal antibody) [13]. Phase II trials of dulanermin in advanced non-small cell lung cancer (NSCLC) combined with chemotherapeutic agents such as paclitaxel, carboplatin, and bevacizumab have shown safety but reported a PR rate of 40% and an overall response rate (ORR) of 40%. However, these results indicated that dulanermin-combined chemotherapy was not superior to chemotherapy alone in patients with NSCLC [29]. Similarly, circularly permuted TRAIL, another rhTRAIL, exhibited poor efficacy in multiple myeloma when combined with thalidomide, achieving an ORR of 22% and a CR rate of 4.9% [30]. TRAIL-receptor (TRAIL-R) targeting agonistic antibodies, including mapatumumab, lexatumumab, conatumumab, and tigatuzumab, have also been evaluated in clinical trials [31–34]. For example, in Phase II trials, the DR4-targeting mapatumumab combined with carboplatin and paclitaxel in patients with advanced NSCLC was safe but failed to yield clinical outcomes [34]. Similarly, DR5-targeting agonistic antibodies such as conatumumab and tigatuzumab showed ORR of 27% and 24.5%, respectively, in advanced NSCLC [32,33]. Notably, DR5-targeting lexatumumab exhibited limited efficacy in solid tumors, with no CR and PR reported in Phase I trials [31]. One of the major challenges in the clinical translation of rhTRAIL is its extremely short half-life (less than 1 h), which leads to insufficient accumulation at target tumor sites [12]. This short half-life is largely attributed to its small molecular size, as therapeutic agents smaller than 5 to 6 nm are known to be rapidly cleared by the kidney [35]. Similarly, monoclonal antibodies targeting TRAIL receptors have demonstrated limited anticancer efficacy in clinical trials due to their inability to effectively initiate apoptosis signaling. TRAIL-induced apoptosis requires trimerization of death receptors (DR4 and DR5) to form a robust DISC [36]. However, the bivalent nature of monoclonal antibodies allows binding to only 2 TRAIL receptors, resulting in incomplete DISC formation and weakened apoptotic signaling [13]. These results collectively highlight the safety profiles of rhTRAIL and TRAIL-R agonistic antibodies in clinical applications while underscoring the limitations in therapeutic efficacy. Insufficient therapeutic levels in TRAIL-mediated clinical trials underscore the necessity for novel delivery strategies to enhance the anticancer efficacy for further development of clinically effective applications. Incorporating biomaterial-mediated delivery techniques represents a promising approach, offering various potential TRAIL formulations and agonistic antibodies. These engineering innovations could extend the circulating half-life and biodistribution of TRAIL, as well as improve its targeted delivery to specific tumor sites.

## Novel TRAIL Delivery Strategies for Cancer Treatments

Among a series of initially developed TRAIL-mediated therapeutics, rhTRAIL and agonistic antibodies targeting DR4 and DR5 have been predominantly utilized. However, their clinical efficacies have been limited, particularly in cancer treatment, leading to the identification of major limitations such as the short half-life of rhTRAIL, inadequate induction of apoptosis by agonistic antibodies, and resistance mechanisms in tumor cells. These challenges have underscored the need for innovative approaches to enhance the bioavailability of TRAIL, increase its activity for greater efficacy, and sensitize TRAIL-resistant cells [13,37,38]. To tackle these issues, various biomaterial-mediated delivery strategies have been implemented to develop efficient TRAIL delivery, with their therapeutic potential evaluated in vivo for future clinical trial applications. Representative TRAIL delivery platforms such as solid NP, gel, and polymeric carrier have been reported for extending the in vivo half-life of TRAIL by enhancing cargo stability [39,40]. Moreover, the conjugation of targeting moieties has been shown to enhance the tumor-specific accumulation of TRAIL, thereby augmenting its antitumor efficacy. Therefore, in this section, we will discuss the advanced and promising approaches developed to enhance the therapeutic potential of TRAIL in cancer treatment, focusing on innovative delivery platforms and strategies designed to address current limitations.

### Solid NPs

An NP-based delivery platform can reduce cytotoxicity and minimize renal clearance, offering substantial advantages for exogenous TRAIL delivery by (a) prolonging its half-life through increased physicochemical stability and (b) enhancing its tumor-targeting capabilities through the conjugation of additional targeting ligands. These advancements render NP-based systems promising candidates for enhancing the efficacy of TRAIL-based cancer therapies [14,41]. The efficacy of solid NP-based TRAIL delivery systems is largely dictated by key physicochemical properties such as size and surface functionalization, which influence stability, biodistribution, and tumor-targeting capability [42,43]. NP size affects circulation time, tumor penetration, and cellular uptake. Particles smaller than 5 to 6 nm are rapidly cleared by the reticuloendothelial system (RES), whereas those within 10 to 200 nm exploit the enhanced permeability and retention (EPR) effect, enabling tumor accumulation [35,44]. Specifically, NPs under 70 nm demonstrate superior tumor accumulation, while those smaller than 20 nm penetrate deeper due to homogeneous distribution and enhanced cell interactions [44–47]. Another crucial factor for improving the functionality of NPs is the functionalization of their surfaces. PEGylation is a widely adopted strategy to enhance the stability and circulatory persistence of NPs. By providing a stealth effect, PEGylation allows NPs to evade clearance by the RES, facilitating enhanced accumulation at tumor sites [48,49]. Additionally, PEGylated superparamagnetic iron oxide NPs introduce reactive groups on the NP surface, which serve as conjugation sites for subsequent functionalization with targeting moieties [50]. Therefore, optimizing the size and surface functionalization of NPs mitigates rapid degradation in the bloodstream and enhances their stability and tumor-specific accumulation of TRAIL, ultimately augmenting its therapeutic efficacy while minimizing off-target effects.

Food and Drug Administration (FDA)-approved iron oxide NPs have been employed as template particulate TRAIL carriers due to their high biocompatibility and superior magnetic properties [51]. Besides their traditional role as contrast agents in magnetic resonance imaging (MRI), recent studies have also explored targeted drug delivery, cell-specific targeting, and multimodal imaging [52–55]. TRAIL conjugated with iron oxide NPs can increase apoptosis in various cancer cells by facilitating accumulation at tumor sites [56,57]. Perlstein et al. [56] demonstrated that conjugating TRAIL to NPs (NP-TRAIL) significantly enhances its apoptotic activity in glioma cells. In both in vitro and in vivo studies, NP-TRAIL effectively targeted and induced apoptosis in glioma cells. This enhanced activity may be attributed to the improved stability and prolonged retention of TRAIL within tumors enabled by NP conjugation. The nanoparticles utilize the EPR effect, allowing NP-TRAIL to preferentially accumulate within tumor tissues, thereby increasing the local concentration of TRAIL and enhancing its interaction with death receptors on glioma cells [58]. Furthermore, iron oxide NP-mediated TRAIL delivery could also initiate the production of reactive oxygen species (ROS) in microenvironments and an increased level of local ROS can up-regulate the expression of DR5 through a ROS/autophagy-dependent pathway. Subsequently, this process leads to resensitization of the TRAIL-mediated apoptosis pathway. Drawing on this mechanism, Shi et al. [57] suggested using TRAIL-conjugated iron oxide NPs (NanoTRAIL) to restore sensitivity and enhance therapeutic efficacy in colorectal cancer. Fluorescence microscopy demonstrated significant ROS generation in colon cancer cells (HT29) treated with NanoTRAIL for 24 h. The addition of *N*-acetylcysteine (20 mM), a ROS scavenger, significantly attenuated ROS production, confirming the role of NanoTRAIL in ROS-mediated mechanisms. The ROS generated by NanoTRAIL facilitated the up-regulation of JNK-autophagy-DR5 signaling pathways, thereby augmenting apoptosis in various colon cancer cells (HCT116, SW480, and HT29). Furthermore, tumor volume monitoring in a xenograft colon cancer model showed tumor suppression (64%) in NanoTRAIL-treated mice compared to controls, highlighting its substantial antitumor efficacy. Biodistribution analysis using a TRAIL ELISA (enzyme-linked immunosorbent assay) kit revealed higher accumulation of NanoTRAIL in tumor tissues compared to free TRAIL protein, suggesting effective targeted delivery and enhanced therapeutic potential. This targeted accumulation was corroborated by increased expression of DR5 proteins in NanoTRAIL-treated tumor samples, further supporting its robust capability to induce tumor cell apoptosis. These findings highlight the promise of NanoTRAIL as an effective therapeutic platform for colorectal cancer, combining ROS-mediated apoptosis induction, targeted delivery, and significant antitumor activity. The rational design of NanoTRAIL leverages FDA-approved iron oxide NPs to sensitize TRAIL-refractory cancers, ultimately enhancing TRAIL-based therapies for clinical application.

NP targeting is achieved through passive and active mechanisms. The EPR effect, a key feature of passive targeting, facilitates NP accumulation in tumors due to leaky vasculature and poor lymphatic drainage [58]. However, passive targeting alone may not completely eliminate off-target effects, particularly in tissues with naturally high vascular permeability. To overcome these limitations, active targeting enhances specificity by functionalizing the surface of NPs with ligands that bind selectively to tumor-specific receptors [59,60] For instance, Duan et al. [59] proposed combining (a) arginine–glycine–aspartic acid (RGD)-TRAIL (for integrin targeting) and (b) a polymeric shell of magnetic microbubbles (MMBs) onto NPs (for increased magnetic resonance contrast and improved diagnostic performance) (i.e., RGD-TRAIL@MMBs). The characteristic binding affinity of RGD toward α_ν_β_3_ and α_ν_β_5_ integrins, which are overexpressed on vascular cells within human tumors, allows the fused RGD-TRAIL protein to specifically target microvascular endothelial cells and enhance apoptosis-inducing activity through caspase-3/8 activation in cancer cells [25]. Furthermore, the designed microbubble system presents a complex multilayer structure: (a) a gas-filled core within the microbubbles improves ultrasound (US) imaging by creating a substantial acoustic impedance difference between the gas inside the microbubble and the surrounding blood, and (b) an SPION-coated shell facilitates MRI and magnetic targeting. This unique configuration allows SPION-coated MMBs to achieve high SPION-loading capacity, strong magnetism, and robust, adjustable enhancement for both US and MRI, making them an effective tool in diagnostic and therapeutic applications [61,62]. Upon administration, TRAIL molecules are delivered to colon cancer cells through NP-mediated endocytosis with the aid of RGD targetability, effectively inducing apoptosis in up to 90% of cancer cells in a dose-dependent manner and inhibiting 46% of tumor growth in tumor xenografts. Consequently, RGD-TRAIL@MMBs serve as a sophisticated molecularly targeted multimodality imaging and delivery system, enhancing both cancer diagnosis and therapy through their integrated targeting and therapeutic capabilities [59].

In addition to the form of recombinant TRAIL protein, TRAIL-encoding plasmid DNA can also be incorporated into NP platforms [60]. Encapsulation of plasmid DNA within NPs ensures sustained delivery of TRAIL to localized tumor sites. In this design, chlorotoxin (CTX) was utilized to enhance targeting efficacy to glioblastoma (GBM) cells, while functionalized chitosan served as a stabilizer to prevent particle agglomeration, thereby improving biodistribution and extending blood half-life [63–65]. It exhibits high binding specificity for various cancer cells, particularly GBM cells. CTX-conjugated iron oxide NPs represent a promising strategy for targeting GBM due to their dual ability to enhance both tumor specificity and blood–brain barrier (BBB) penetration [63,66] GBM cells overexpress matrix metalloproteinase-2 (MMP-2), an enzyme involved in the degradation of extracellular matrix components that facilitates tumor invasion and metastasis. CTX binds with high affinity to MMP-2 isoforms present on GBM cells, allowing for specific tumor localization [63]. CTX can exploit receptor-mediated transcytosis pathways to cross the BBB. Its interaction with MMP-2 not only aids in tumor targeting but also facilitates transcytosis across the endothelial cells of the BBB, enhancing delivery to brain tissue [64,66]. Therefore, systemic administration of TRAIL-CTX-coated iron oxide NPs to mice bearing T98G-derived xenografts significantly inhibited tumor growth, achieving 78% tumor suppression, and effectively induced apoptosis in tumor tissues. Since TRAIL can be released as a soluble ligand, these findings indicate that TRAIL-encoding plasmid DNA-conjugated NPs were successfully delivered to tumors, enabling localized TRAIL expression and promoting apoptosis in both transfected cells and neighboring cells through the secretion of soluble TRAIL.

**Fig. 2.**
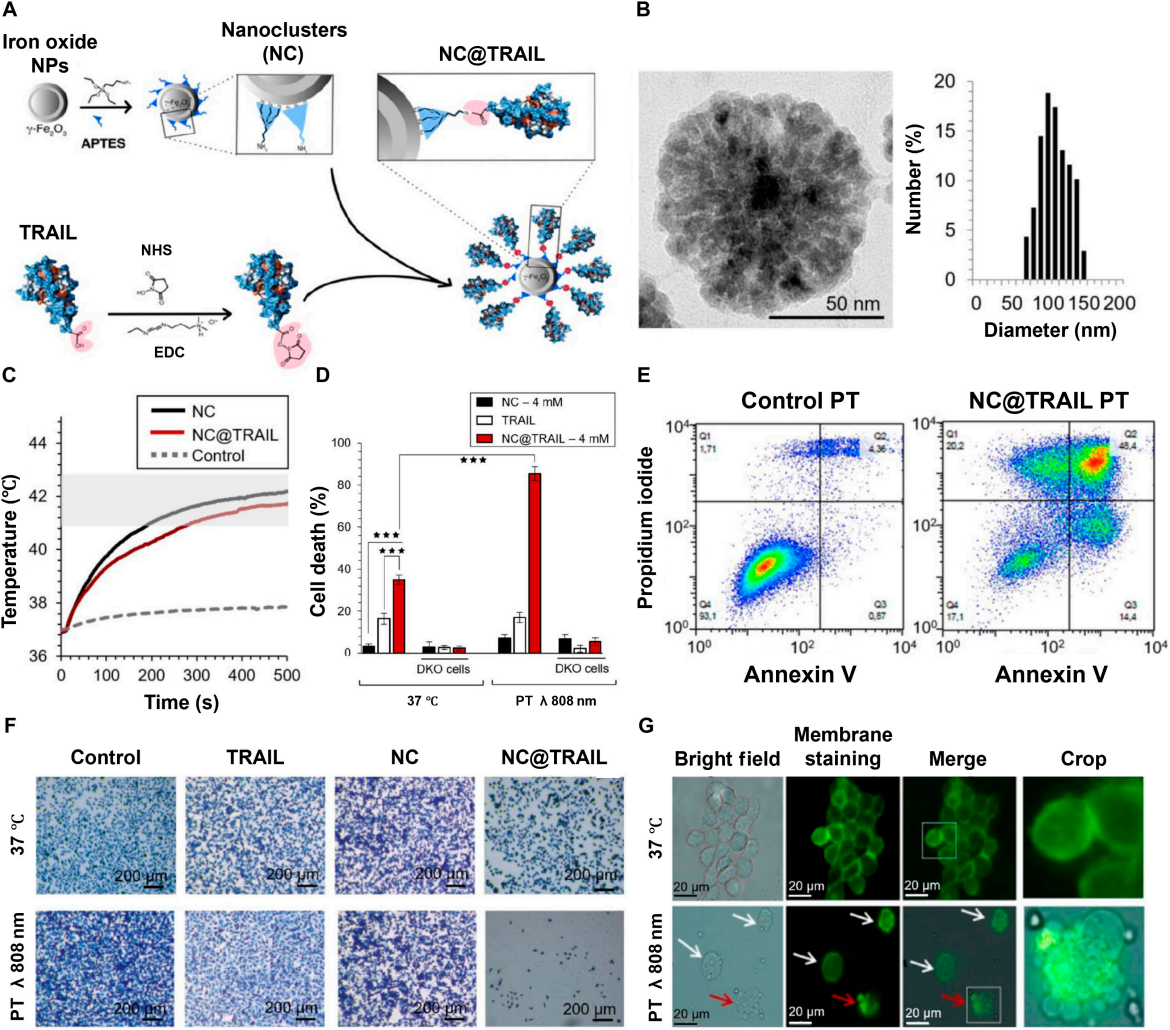
Synergistic effects of NC@TRAIL and photothermal therapy (PT) in breast cancer cells. (A) Schematic illustration of the functionalization of iron oxide nanoclusters (NCs) and the conjugation of TRAIL onto NCs. (B) Transmission electron microscopy (TEM) images and size distribution analysis of iron oxide NCs. (C) Temperature increment for NC@TRAIL and NCs with MDA-MB-231 cancer cells in PT mode. (D) Cell death in MDA-MB-231 cells and TRAIL-receptor-deficient (DKO) cells treated with TRAIL alone, NCs, and NC@TRAIL at 37 °C and under PT mode. (E) Assessment of early and late apoptosis and/or necrosis using annexin V and propidium iodide staining following PT treatment. (F) Cell viability in MDA-MB-231 cells determined by methylene blue staining (blue indicates viable cells and gray-white indicates cell death) across various treatment groups: control, TRAIL alone, NCs, and NC@TRAIL at 37 °C and under PT mode. (G) Bright-field microscopy and green membrane staining of MDA-MB-231 cells, highlighting the effects of NC@TRAIL under PT treatment. White arrows indicate cell membrane fragility compared to the well-defined membranes of untreated cells; red arrows show lysosomal membrane disruption caused by NC@TRAIL interacting with agonist death receptors, ultimately leading to cell death. (Figures were reproduced with copyright permission from Ref. [69].)

Another notable characteristic of iron oxide NPs is their capacity to generate heat through both magnetothermal (MHT) and photothermal (PT) processes [67,68]. For instance, TRAIL-conjugated iron oxide nanoclusters (NC@TRAIL) have enhanced pro-apoptotic activities against breast cancer cells through NP-mediated MHT or PT processes [69]. The functionalization and TRAIL conjugation of iron oxide nanoclusters (NCs) are achieved through coupling reactions between the amino groups of (3-aminopropyl)triethoxysilane-functionalized iron oxide NPs and the carboxyl groups of the TRAIL protein (Fig. 2A). Transmission electron microscopy revealed well-dispersed iron oxide NCs with a uniform size distribution (104 ± 20 nm), suggesting their suitability for biomedical applications while minimizing renal clearance possibility (Fig. 2B). The synergistic effects of NC@TRAIL and PT were evident in the temperature increment observed during PT treatment, which demonstrated efficient heat generation in MDA-MB-231 breast cancer cells (Fig. 2C). The combination of NC@TRAIL and PT significantly enhanced cell death compared to TRAIL or NCs alone, with minimal effects in TRAIL-receptor-deficient (DKO) cells. This finding suggested that NC@TRAIL specifically interacts with TRAIL receptors DR4 and DR5 in MDA-MB-231 cells, inducing cancer cell death during thermal treatment (Fig. 2D). Early and late apoptosis were further confirmed through annexin V and propidium iodide staining, which demonstrated enhanced cell death in NC@TRAIL-treated cells under PT mode (Fig. 2E). Additionally, the combination of NC@TRAIL and PT demonstrated substantial therapeutic potential, as evidenced by a dramatic reduction in viable cells compared to controls or individual treatments (Fig. 2F). This underscores the enhanced efficacy achieved by integrating TRAIL delivery with PT, providing a synergistic effect in targeting cancer cells. Furthermore, the activation of NC@TRAIL by either MHT or PT processes induced localized thermal hotspots near DR targets, resulting in membrane disruption and subsequent cancer cell death (Fig. 2G). These findings highlight the promising application of thermal nanoheaters in achieving targeted apoptosis and advancing remote-controlled, tumor-specific thermal therapies.

Collectively, iron oxide NPs represent a promising platform for TRAIL delivery due to their (a) unique physicochemical properties, (b) facile surface functionalization with multiple components, (c) prolonged circulation half-life, and (d) enhanced tumor-targeting capability. Despite these advantages, certain challenges remain, including (a) nonspecific accumulation in the liver and spleen and (b) an unclear clearance mechanism from the body. The following section explores a polymer-based TRAIL delivery platform with a different approach.

### Gel-based strategies

As previously discussed, iron oxide NPs have been extensively investigated for their use in phototherapy applications in cancer treatment, encompassing PT therapy and photodynamic therapies. Although these phototherapies have demonstrated enhanced anticancer efficacy, their clinical application is hindered by several challenges. The most significant limitation is the restricted penetration depth of light, which leads to incomplete treatment of tumors located beyond the irradiation range. This incomplete elimination of cancer cells can lead to residual cancer cells, heightening the risk of tumor recurrence and metastasis. Additionally, even minor mistargeting of PT agents can cause thermal damage to adjacent normal tissues [70,71]. To address these challenges, encapsulating TRAIL within carriers such as coacervates (Coas) or hydrogels and subsequently adding cancer-targeting moieties offers a viable alternative to directly immobilizing TRAIL on NPs. This strategy not only facilitates local delivery of TRAIL to tumors but also enhances its protection. Although TRAIL exhibits effective affinity toward TRAIL receptors on cancer cell membranes, its short in vivo half-life, particularly in the soluble form, remains an important challenge in clinical development [72]. Hence, a series of encapsulation techniques using functional biomaterials have been developed to preserve the biological activity of TRAIL and to achieve synergistic effects from delivered TRAIL and other functional moieties.

**Fig. 3.**
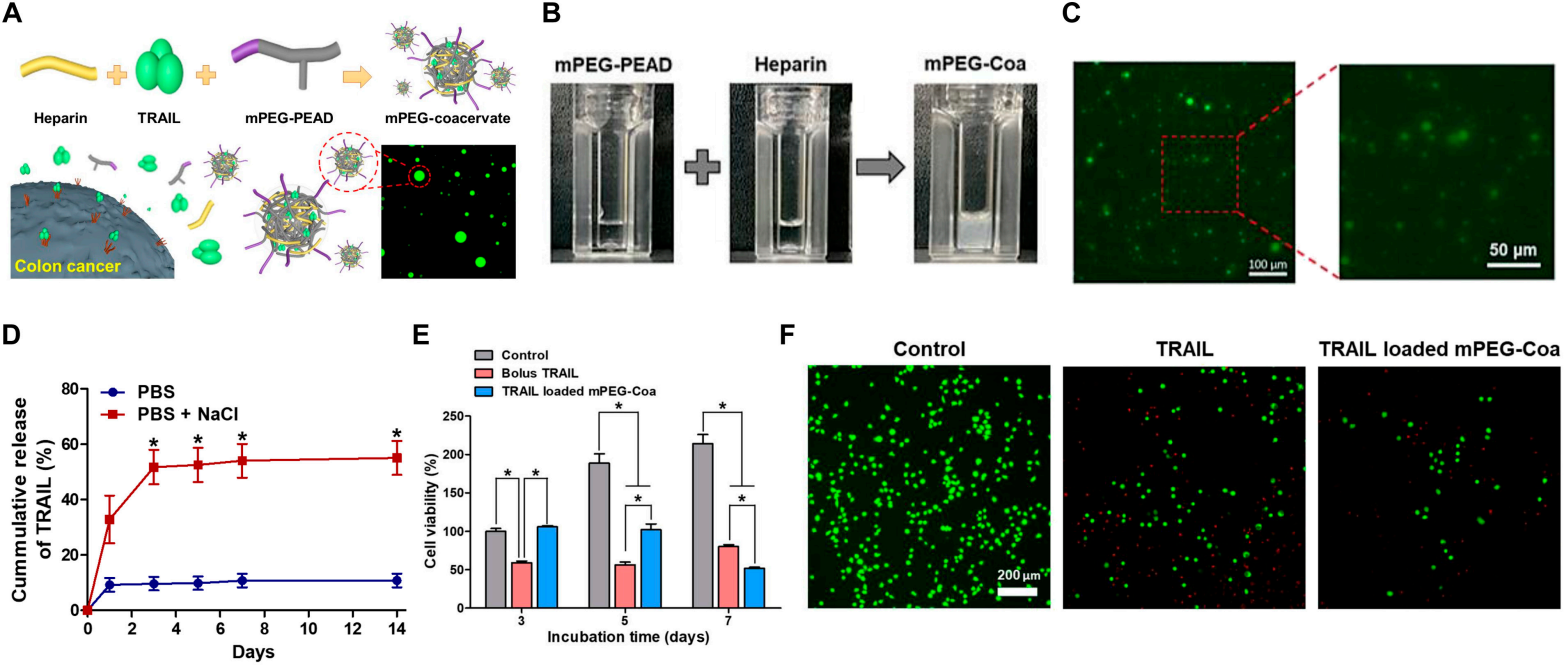
Exogeneous TRAIL delivery using mPEG-Coa to prevent colon cancer recurrence. (A) Fabrication process of mPEG-Coa loaded with TRAIL and mechanism for inducing cancer cell apoptosis. (B) Macroscopic observation of coacervation using mPEG-PEAD, heparin, and mPEG-Coa (i.e., liquid–liquid phase separation) and (C) fluorescence photographs of mPEG-Coa. (D) Release profile of TRAIL from mPEG-Coa. (E) Viability of HCT-116 colon cancer cells after treatment for 3, 5, and 7 days. (F) Fluorescence microscope images of live/dead after 7 days of treatment. (Figures were reproduced with copyright permission from Ref. [80].)

Coa is a self-assembled spherical polyelectrolyte complex formed via electrostatic interactions between a polycation and a polyanion in an aqueous environment [73]. Among various Coa formulations, poly(ethylene arginylaspartate diglyceride) (PEAD)-based Coa has been employed due to its improved biocompatibility over conventional polycations and its capacity for controlled cargo release [74]. This platform has been widely utilized for the delivery of growth factors in diverse tissue engineering applications, including osteochondral tissue regeneration [75], vascular regeneration [76], bone regeneration [77], and skin tissue repair [78,79]. In addition to their effectiveness in tissue engineering, the selective drug release capabilities of Coa in the TME have significantly expanded their application in anticancer drug delivery, positioning them as a promising platform for cancer treatment. Leveraging this phenomenon, methoxy-poly(ethylene glycol)-conjugated Coas (mPEG-Coas) were developed by coassembling mPEG-PEAD and heparin to achieve stable TRAIL encapsulation and efficient therapeutic delivery (Fig. 3A) [80]. Upon mixing mPEG-PEAD and heparin solutions, coacervation was immediately observed, as evidenced by the transition to a turbid solution indicative of liquid–liquid phase separation (Fig. 3B). The synthesized mPEG-Coa maintained a stable spherical morphology (Fig. 3C), with an average particle size of 491.4 nm. Moreover, mPEG-Coas exhibited high TRAIL loading efficiency (>88%) and a slow TRAIL release rate of approximately 10.9% over 14 days in a normal PBS environment. In contrast, the high sodium concentration characteristic of the TME (i.e., PBS with 185 mM NaCl) triggered Coa dissociation, leading to a burst release of approximately 59.4% over 14 days (Fig. 3D). Notably, in proteolytic stability assays, TRAIL-loaded mPEG-Coa retained 58.6% of TRAIL bioactivity following trypsin treatment, compared to only 31% for unprotected bolus TRAIL, highlighting its protective capability. Consequently, Coa-mediated TRAIL delivery significantly suppressed the proliferation of HCT-116 colon cancer cells, achieving ~50% inhibition after 7 days of treatment, compared to ~150% proliferation in the untreated control group (Fig. 3E and F). These findings underscore the potential of Coa-based systems as an advanced platform for TRAIL delivery, offering enhanced protection and prolonged therapeutic efficacy in cancer treatment.

Hydrogel also serves as a TRAIL-encapsulating carrier for cargo protection, and a reservoir for additional incorporation using other functional moieties for enhanced anticancer effects. This flexible, crosslinked polymer network, formed by hydrophilic macromonomers, effectively protects cargo TRAIL from harsh physiological conditions such as enzymatic degradation and immune responses [81,82]. The softness and flexibility of hydrogels improve their biocompatibility with native tissues, thereby minimizing the risk of immune reactions [83]. Additionally, various bioactive agents can be incorporated into the hydrogel matrix, enabling combinatorial therapies with delivered TRAIL [84,85]. Owing to their tunable physical properties, hydrogels can facilitate the controlled release of encapsulated agents through degradation triggered by specific environmental cues. For instance, (a) an acidic environment typically accelerates hydrolysis, enhancing cargo release, and (b) high-energy ultraviolet (UV) light induces microgel degradation by cleaving *o*nitrobenzyl ether moieties in hydrogel, accelerating the release of encapsulated proteins. Moreover, even low-energy near-infrared (NIR) light can remotely control hydrogel deformation by utilizing upconversion NPs, which convert multiple NIR photons into a single UV photon, triggering localized degradation and controlled drug release [86]. These dynamic and responsive properties enable precisely tuned cargo release from the hydrogel, ultimately enhancing the tumor inhibition effect.

**Fig. 4.**
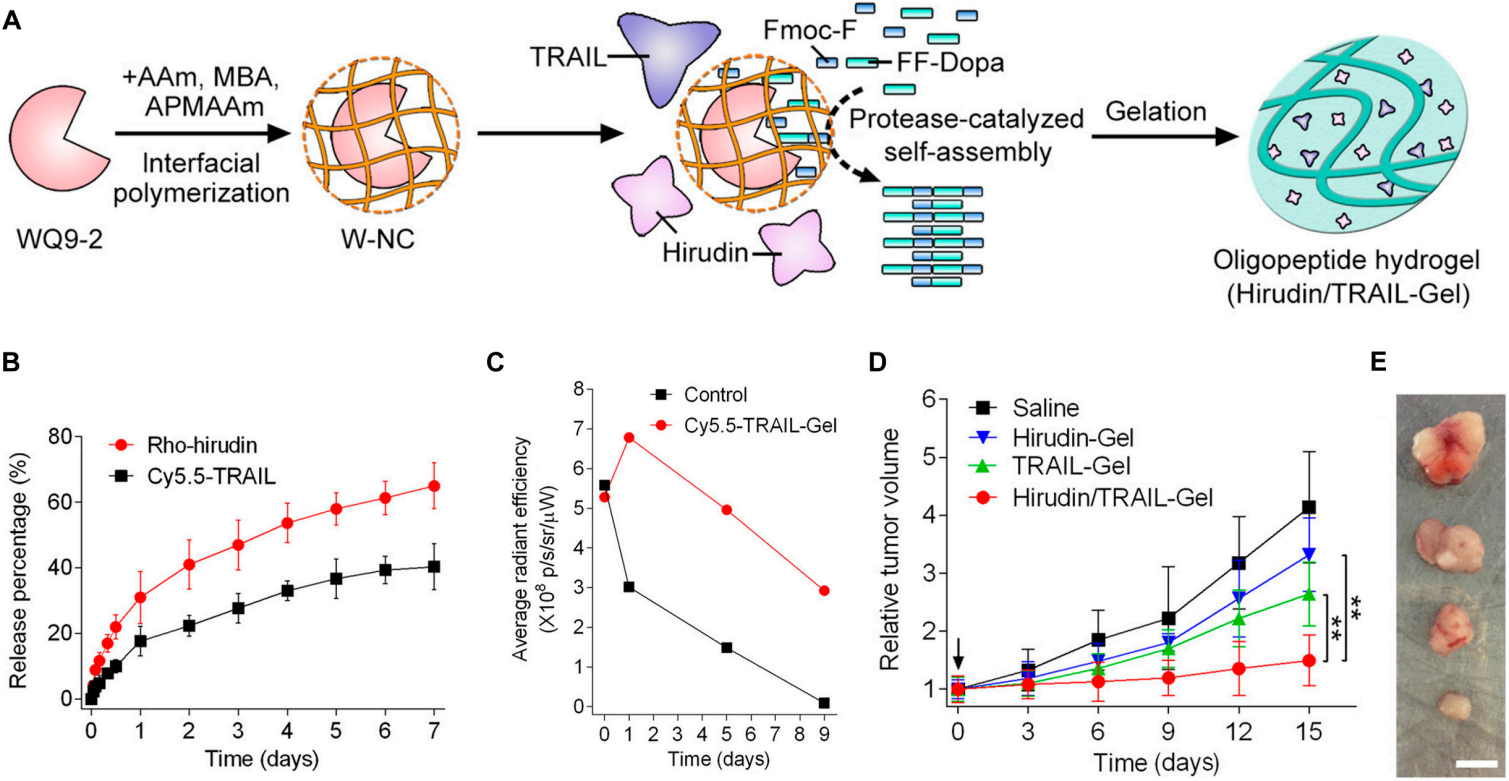
Hydrogel-encapsulated TRAIL for effective suppression of triple-negative breast cancer. Substrate-selective protease-catalyzed self-assembly of the oligopeptide hydrogel. (A) Schematic representation of the fabrication method for the oligopeptide hydrogel. (B) Release profiles of both protein therapeutics from the oligopeptide hydrogel at pH 7.4. (C) Analysis of the tumor site after administration for each group. (D) Relative tumor volume comparison after treatment with Hirudin/TRAIL-Gel and (E) tumor volume at 15 days. Scale bar: 0.5 cm. (Figures were reproduced with copyright permission from Ref. [87].)

For instance, codelivery of TRAIL and Hirudin encapsulated in hydrogels exhibited a synergistic therapeutic effect, combining Hirudin-mediated anti-angiogenesis with TRAIL-induced apoptosis in a triple-negative breast cancer model [87]. To achieve in situ interfacial polymerization of the hydrogel matrix, a zinc metalloprotease (WQ9-2) was encapsulated within a single-protein nanocapsule (W-NC). This nanocapsule, synthesized through the interfacial polymerization process, preserved the proteolytic activity of WQ9-2 while minimizing unintended interactions with surrounding proteins. The hydrogel precursors, Fmoc-Phe (Fmoc-F) and Phe-Phe-Dopa (FF-Dopa), reacted with the W-NC to form Fmoc-FFF-Dopa. This reaction facilitated the self-assembly of the hydrogel system, which was subsequently coloaded with TRAIL and Hirudin (Hirudin/TRAIL-Gel) (Fig. 4A), leading to sustained release of cargo even at pH 7.4. Hirudin, an antiangiogenic protein, directly inhibited angiogenesis by acting on thrombin, which can be used to block the formation of intratumoral blood vessels. The released Hirudin and TRAIL from the Hirudin/TRAIL-Gel led to a significantly enhanced apoptotic ratio (40%) in MDA-MB-231 cells, compared to the TRAIL-encapsulated hydrogel alone (Fig. 4B). In MDA-MB-231 tumor-bearing mouse models, prolonged retention of TRAIL and Hirudin, released from the Hirudin/TRAIL-Gel, was observed at the tumor site. Subsequently, complete tumor removal was achieved within 5 days in the Hirudin/TRAIL-Gel group, whereas tumors persisted for 9 days in the group treated with TRAIL-Gel alone (Fig. 4C). Tumor growth was observed over time following treatment (Fig. 4D), with the smallest tumor observed and harvested from mice 15 days post-administration (Fig. 4E). Therefore, this study demonstrated that hydrogel-based encapsulation enhanced the therapeutic outcomes of combinatorial delivery of TRAIL with the coadministered drug.

The modification of nanogel surfaces can enhance cancer-targeting capabilities through the decoration of cancer-targeting moieties. For example, a tumor stroma-targeting nitric oxide nanogel (LQT-TRAIL-NO@Nanogel) was developed to improve the localization of TRAIL to a target region and controlled release under acidic conditions [23]. Briefly, the core of nanogel was fabricated through a water–oil single emulsion process using silk fibroin (SF) and phospholipid DOPA. The SF nanogel was then coated with lipids containing DSPE-PEG_2k_-maleimide, which enabled the conjugation of the LQT28 peptide via maleimide-thiol reaction between the DSPE-PEG_2k_-maleimide and the thiol group of the peptide. The LQT28 peptide, a tumor stroma-targeting moiety, was conjugated to the nanogel surface, achieving the increased binding specificity compared to other peptide candidates such as RDY56 or FSV117. This conjugation resulted in heightened accumulation within tumors, highlighting the effectiveness of the LQT28 peptide in enhancing targeted delivery. The primary components of the nanogel included the following: (a) a lipid-poly(lactic-co-glycolic) acid NPs shell, encapsulating therapeutic agents including a synthetic nitric oxide (NO) donor dinitrosyl iron complex, anticancer drug, and TRAIL-loaded hydrogel; (b) a TRAIL-loaded hydrogel, comprising an SF hydrogel core designed to carry the TRAIL cargo; and (c) NO, activating apoptotic pathways by modulating anti-apoptotic BCL-2 family members and tumor suppressor p53, thereby migrating tissue fibrosis in pancreatic ductal adenocarcinoma (PDAC). Systemic injection of 143-nm nanogels into orthotopic PDAC models effectively codelivered both NO and TRAIL to the targeted stromal regions, exhibiting a significantly enhanced anticancer effect. This resulted in a significant reduction in PDAC tumor volumes to approximately 50 mm^3^ after 16 days of treatment, compared to about 200 mm^3^ in the control group. Therefore, the targeted delivery of NO reduced tumor desmoplasia, thereby improving TRAIL penetration into the tumor core and demonstrating the enhanced antitumor efficacy of the TRAIL-based combinatorial therapy, leading to a promising approach for cancer treatment. Table S2 summarizes the fabrication method, tumor models, antitumor mechanisms, and therapeutic effects of biomaterial-based TRAIL delivery.

The encapsulation techniques using colloidal and hydrogel platforms provide several advantages, including (a) local delivery of TRAIL to targeting regions, (b) protection of TRAIL’s bioactivity in harsh environments, (c) provision of sustained TRAIL release from the gel, (d) good biocompatibility, and (e) expecting synergistic effects by incorporating with other therapeutic reagents. Modifying the gel surfaces with tumor-targeting moieties further facilitates tumor-specific TRAIL delivery. This approach is promising in cases where intratumoral administration is challenging due to local progression or the small size of the tumor. Precisely regulated size control in TRAIL-embedding colloidal or gel platforms also enables systemic administration, while conventional injectable hydrogels are commonly applied via intratumoral administration.

## Cell-Based Carrier for TRAIL Delivery

In the previous section, we introduced the material-mediated anticancer strategies with phototherapy or chemotherapy. To advance this technology, researchers have designed various materials capable of specifically recognizing cancer cells or responding to the acidic pH of the TME. Another approach to identifying cancer cells involves utilizing the major histocompatibility class I (MHC-I) complex, which is present on most cells in the body and plays a critical role in distinguishing normal cells from abnormal ones [88,89]. The structure of the MHC-I complex is heterodimers composed of the polymorphic heavy chain and the light β2-microglobulin chain. By displaying antigenic peptides on the cell surface, the MHC- I complex facilitates scanning by the T-cell receptors. This interaction allows CD8^+^ T cells to recognize the presented peptides and eliminate cancerous or infected cells [90,91].

Immune cells play a pivotal role in distinguishing healthy cells from cancer cells, enabling them to selectively recognize and eliminate abnormal cells. For instance, natural killer cells can target cancer cells that have lost MHC molecule [92] while macrophages [89] and T cells contribute to the elimination of cancer cells through their ability to identify lost or damaged MHC molecules [93]. The delivery of TRAIL on the cell surface offers distinct advantages over conventional cell–drug conjugates. While immune cell surface functionalization with chemotherapeutic agents has demonstrated potential in enhancing targeted drug delivery and augmenting antitumor efficacy, several challenges remain. These include (a) heterogeneous coating of chemotherapeutic agents on the cell surface, (b) reliance on the directionality of drug release from effector cells to target cells, and (c) the unintended internalization of these agents into carrier cells, which may result in cytotoxicity and impair the therapeutic functionality of the immune cells [94,95]. In contrast, TRAIL selectively induces extrinsic apoptosis by binding to specific death receptors predominantly expressed on tumor cell surfaces, whereas normal cells express decoy receptors that inhibit apoptotic signaling [8]. By decorating cell surfaces with TRAIL, the delivery system harnesses active targeting via receptor–ligand interactions, ensuring high local concentrations of TRAIL at tumor sites while minimizing systemic exposure. In this regard, cell-based TRAIL delivery strategies showed great promise for targeted cancer cell elimination, minimizing off-target effects and reducing the risk of damage to healthy tissues [88,89].

A significant challenge in cancer treatment remains the prevention of tumor metastasis. Metastasis, the process by which cancer spreads and secondary tumors develop at sites distant from the original tumor, presents significant challenges in current cancer treatments and is a leading cause of cancer-related deaths, and the detailed underlying mechanism remains poorly elucidated [96]. Large numbers of cancer cells are released into circulation in the bodies of cancer patients, and circulating tumor cells (CTCs) persist in the bloodstream, facilitating migration to other sites [97,98]. Even a few residual CTCs can spread, leading to secondary tumors [99,100]. This characteristic is a hallmark of cancer malignancy, with metastasis responsible for more than 90% of cancer-related deaths [101]. To develop effective technologies targeting CTCs, therapeutic agents could remain stable and functional in the bloodstream. In this respect, cell-based carriers present a good option. Therefore, in this section, we explore technologies aimed at eliminating both solid cancer cells and CTCs. We highlight recent advancements in (a) genetically engineered cellular products designed to release TRAIL effectively and induce apoptosis by binding to receptors solid cancers, and (b) biomaterial-mediated transplantable cell surface modification using liposomes to enhance TRAIL function, ultimately targeting CTCs.

### Genetically engineered living cells

As described in the “Current Status of TRAIL Therapy in Clinical Trials” section, TRAIL-mediated anticancer treatment is facilitated by secreted TRAIL, functioning as a protein drug. One common approach in immune cell engineering involves genetically modifying cells to express key signaling ligands and receptors, thereby enhancing therapeutic efficacy against solid cancers. Among various cell types, genetically modulated macrophages have shown enhanced tumor suppression through facilitated TRAIL binding cascades, in addition to their multiple therapeutic functions such as phagocytosis, cytokine secretion, and antigen presentation [102,103].

For example, Zhu et al. have developed surface-engineered macrophages capable of trimeric TRAIL (Tri-TRAIL-iM) through induction by a tumor-conditioned specific promoter, Arg1, within the TME. In the vector design, the TRAIL cDNA sequence was cloned into the lentiviral vector pCDH, which also includes the human C-peptide of α1(I) collagen, resulting in the construct pCDH-MCSV-Tri-TRAIL. This method leads to the formation of a disulfide bond-linked homotrimer by in-frame fusion with the C-terminus of TRAIL [17]. Additionally, the MCSV promoter was replaced with the Arg1 promoter (pCDH-Arg1-Tri-TRAIL), enabling a targeted response to tumor-associated stimuli due to elevated Arg1 expression in RAW264.7 cells. Under normal culture conditions, low levels of TRAIL were detected. On the other hand, elevated TRAIL levels were observed in Tri-TRAIL-iM following exposure to tumor-conditioned medium. Leveraging this tumor-responsive release, Tri-TRAIL-iM demonstrated highly specific anticancer activities across multiple cell lines, including breast cancer (4T1 cells), melanoma (B16 cells), and colon cancer (CT26 cells), under in vitro and in vivo (4T1 tumor-bearing mouse models) conditions [104].

Another promising approach to enhancing anticancer effects through living cells involves using mesenchymal stem cells (MSCs). MSCs are extensively utilized in therapeutic delivery platforms due to their intrinsic low immunogenicity. Specifically, MSCs inherently lack the expression of human leukocyte antigen-DR (HLA-DR) and critical costimulatory molecules, including CD80, CD86, and CD40, which are typically required for the activation of T and B lymphocytes, contributing to the low immunogenicity of MSCs [105,106]. This absence of immunogenic markers significantly reduces the host immune rejection, making MSCs highly suitable for allogeneic transplantation without provoking robust immune responses [106]. These unique properties underscore the potential of MSCs as ideal living cell carriers for TRAIL delivery, combining low immunogenic risk with enhanced therapeutic modulation within the TME. Moreover, due to their intrinsic migratory ability toward injury sites, MSCs offer a unique opportunity to reach tumor sites. Notably, MSCs have been observed migrating into tumor tissue similarly to their homing response to injured tissues in the TME [107]. Among various signaling pathways, the CXCL12/CXCR4 axis has been extensively studied for MSC migration to the TME [108]. One of the primary challenges in developing effective cancer therapies using MSCs is their limited tumor specificity. To enhance cancer killing at the tumor site, MSCs have been transfected with a plasmid encoding a secretable form of TRAIL, effectively inhibiting the proliferation of rat glioma (C6) cells. By harnessing the tumor-homing ability of MSCs, the transfected cells acquired TRAIL as a tumor-specific cytotoxic agent, enabling targeted treatment of malignant gliomas. Once the engineered MSCs reached the TME, TRAIL was released from them by interacting with death receptors on the tumor cells. Through this mechanism, TRAIL-transfected MSCs significantly inhibited C6 cell growth, with an inhibition rate of 16.7% after 1 day of coculture, compared to 63.7% in control C6 cells treated with a control plasmid. These findings underscore the potential of using MSCs as anticancer agents to treat one of the most refractory cancers [109].

While stem cell therapy shows promise in anticancer treatment, several limitations should be addressed. First, enhancing MSC migration to tumor sites requires activated inflammatory responses to improve MSC homing capacity. Second, the absence of MHC class I expression on stem cells renders them targets for natural killer cells, posing an additional challenge for future applications. Further studies are necessary to address these limitations and explore the integration of stem cells in cancer immunotherapy.

### Liposome attachment on living cell surface

While genetic modification has been widely utilized to enhance TRAIL secretion in living cells, unexpected tumorigenesis may result from the use of viral vectors, and transfection efficiency can be difficult to precisely control along with the cell-type-dependent variation [110,111]. As a notable breakthrough, biomaterial-based cell surface engineering technology provides an alternative by modifying cellular membranes without disrupting the intrinsic functionalities of cells or manipulating genetic characteristics. Cell surface modification strategies utilizing biomaterials, including glycoengineering with click chemistry, direct chemical conjugation, and lipid-based hydrophobic interactions, have been widely explored. These approaches have several key limitations inherent to these techniques. For instance, metabolic glycoengineering enables MSCs to express azide groups on their surfaces, allowing binding to dibenzylcyclooctyne (DBCO)-functionalized therapeutic agents through bio-orthogonal click chemistry for targeted tumor interactions. However, glycoengineering is time-consuming (requiring ~48 h) and dependent on unpredictable cellular metabolic processes [112]. Furthermore, direct chemical conjugation methods involve covalently attaching therapeutic agents to cell surfaces, often resulting in heterogeneous modification due to random conjugation sites and the potential for irreversible alterations to the cell membrane, which may affect cell functionality [113]. Alternatively, lipid-based biomaterials offer a promising option for cell surface modification. Hydrophobic lipid anchors are nontoxic to cells and do not compromise the intrinsic properties of the cells [114,115]. Recent studies demonstrated that lipid-based biomaterials can uniformly coat cell surfaces within 30 min while preserving key cellular functions [110,111,116]. Therefore, lipid-based biomaterials could represent a convenient, efficient, and biocompatible alternative to traditional surface modification methods, potentially overcoming the limitations of current engineered cell therapies.

**Fig. 5.**
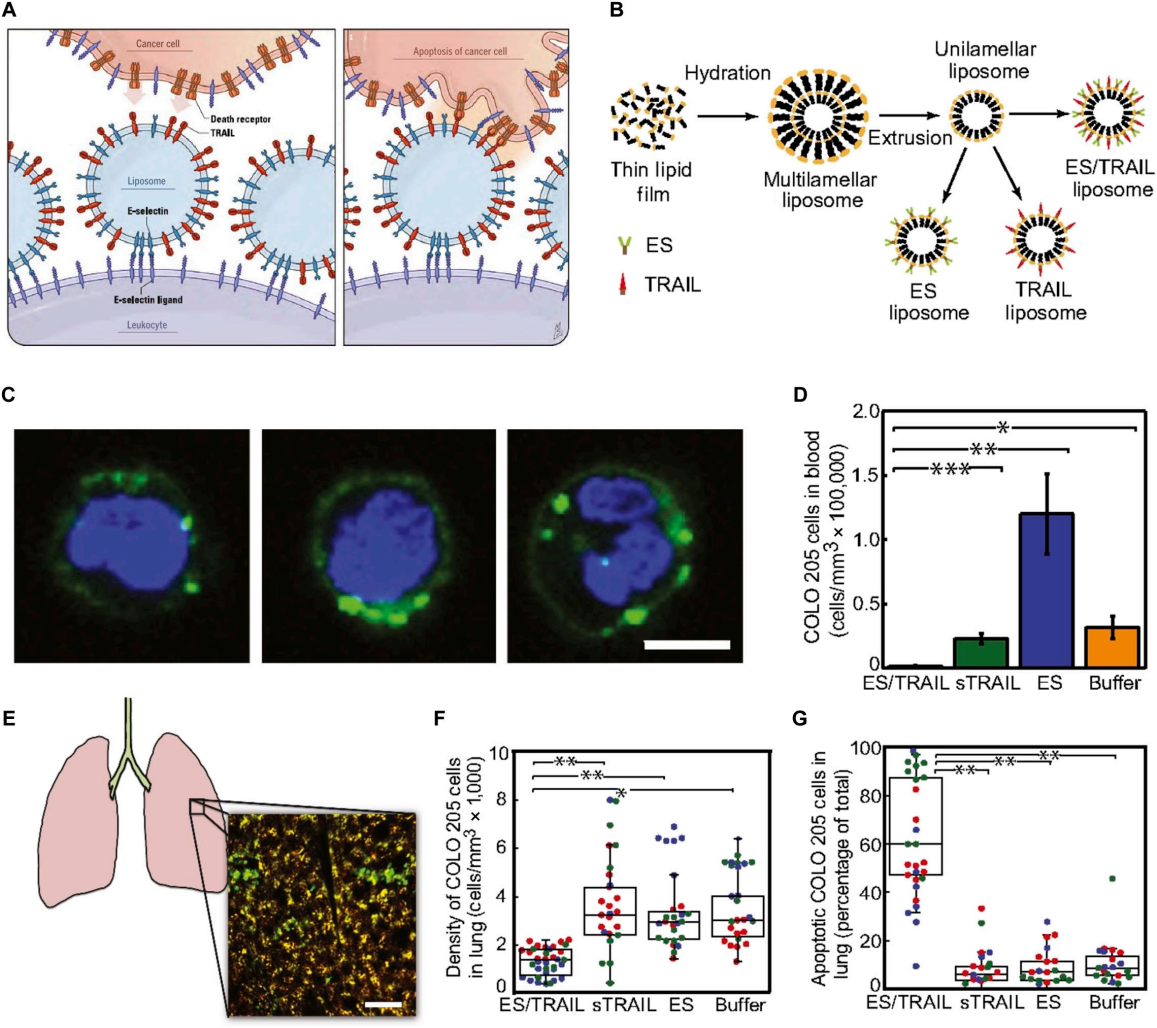
Liposome-mediated TRAIL delivery for the prevention of cancer metastasis. (A) Mechanism of surface-decorated leukocytes with liposomes interacting with the death receptors on circulating tumor cells. (B) Fabrication method of ES/TRAIL liposomes. (C) Confocal images showing ES/TFRAIL-liposomes (green) attached to leukocytes (blue). Scale bar: 5 μm. (D) Number of surviving cancer cells recovered per volume of blood in mice post-administration. (E) Image of mouse lung alongside a 2-photon excited fluorescence (2PEF) image stack. Hoechst-labeled COLO 205 cells (green) are shown suspended within lung tissue, visualized by autofluorescence (yellow). Scale bar: 80 μm. (F) Density and (G) apoptosis of COLO 205 cells in the lung for each experimental group. (Figures were reproduced with copyright permission from Ref. [119].)

To engineer the cellular membrane surfaces with TRAIL, liposomes can be utilized to incorporate the immune cells. Consequently, surface modification of liposome with additional cancer targeting ligands such as TRAIL could significantly augment their precise cancer targeting ability. The incorporation of TRAIL into liposomes extends their circulation time in vivo, improving their therapeutic potential [117,118] For example, dual compartments of E-selectin (ES) and TRAIL were decorated onto liposome surfaces to enhance adherence to leukocyte membranes via ES and targeted cancer cell elimination via TRAIL (Fig. 5A). Liposomes, fabricated with nickel nitrilotriacetic acid (Ni-NTA)-conjugated lipids, were designed to interact with his-tagged TRAIL and his-tagged ES through Ni-NTA and his-tag, resulting in the formation of ES/TRAIL liposomes (Fig. 5B). The ES component allowed the ES/TRAIL liposomes to bind to leukocytes under physiological shear flow conditions (shear rate: 188 s^−1^), recreating the physical forces observed in vitro. Within the postcapillary venule, where selectin-mediated adhesion occurs, shear rates promote interactions between circulating cells and the endothelial cell wall (Fig. 5C). The surface engineering of leukocytes with ES/TRAIL liposomes did not induce significant leukocyte death. Furthermore, these engineered leukocytes exhibited no off-target effects on normal endothelial cells, highlighting their excellent biocompatibility and potential for in vivo safety. Under these shear flow conditions, ES/TRAIL therapy demonstrated a more potent cancer-killing effect against COLO 205 colorectal cancer and PC-3 prostate cancer cells compared to static buffer conditions. Notably, leukocytes decorated with ES/TRAIL liposomes showed enhanced cancer-killing effects against COLO 205 and PC-3 cells compared to leukocytes functionalized with only ES liposomes. Cancer cell elimination was quantified by counting viable COLO 205 and PC-3 cells per blood volume (Fig. 5D). The interaction between ES/TRAIL liposomes and CTCs was further assessed in vivo by injecting ES/TRAIL liposomes into mice 2 h after the tail-vein administration of COLO 205 cells. The number of viable cancer cells demonstrated the dual-targeting efficacy of ES/TRAIL liposomes (<2,000 cells/ml of blood), showing improved therapeutic performance compared to control ES liposomes (~130,000 cells/ml of blood). Subsequently, the apoptotic effects of ES/liposomes on cancer cells were evaluated in the mouse lung (Fig. 5E). The results demonstrated that the ES/TRAIL liposome-treated group exhibited a reduction in cancer cell density and a significant increase in cancer cell apoptosis (Fig. 5F and G) [119].

Similarly, ES/TRAIL-functionalized liposomes (ETLs) were explored as a strategy to migrate metastasis in aggressive triple-negative breast cancer cells. ETL was prepared using a similar fabrication process that involved the interaction between his-tagged proteins (ES and TRAIL) and Ni-NTA for efficient functionalization. Under shear flow conditions (shear rate of 150 s^−1^), ETL treatment led to a reduced percentage of viable cells (46%) compared to the soluble form of TRAIL (ST) and naked liposome (NL) groups, which demonstrated 87% and 86% viability, respectively. To further assess the reduction in metastasis burden, a 4T1 breast carcinoma model was developed, focusing specifically on neutralizing metastasis after surgical removal of the primary breast tumor. After systemic injection of 3 different types of liposomes (ETL, ST, and NL), followed by 3 additional doses over a 48-h period, the ETL group exhibited a significantly enhanced anti-metastatic effect (2-fold reduction) compared to the ST and NL groups. This elimination was observed after surgery (day 20 and subsequent administration of 2 additional doses and 3 more injections of ETL, ST, or NL). The improved therapeutic outcome was attributed to targeted delivery facilitated by ES binding to leukocytes, thereby enabling more efficient in vivo targeting of circulating cancer cells [120].

**Fig. 6.**
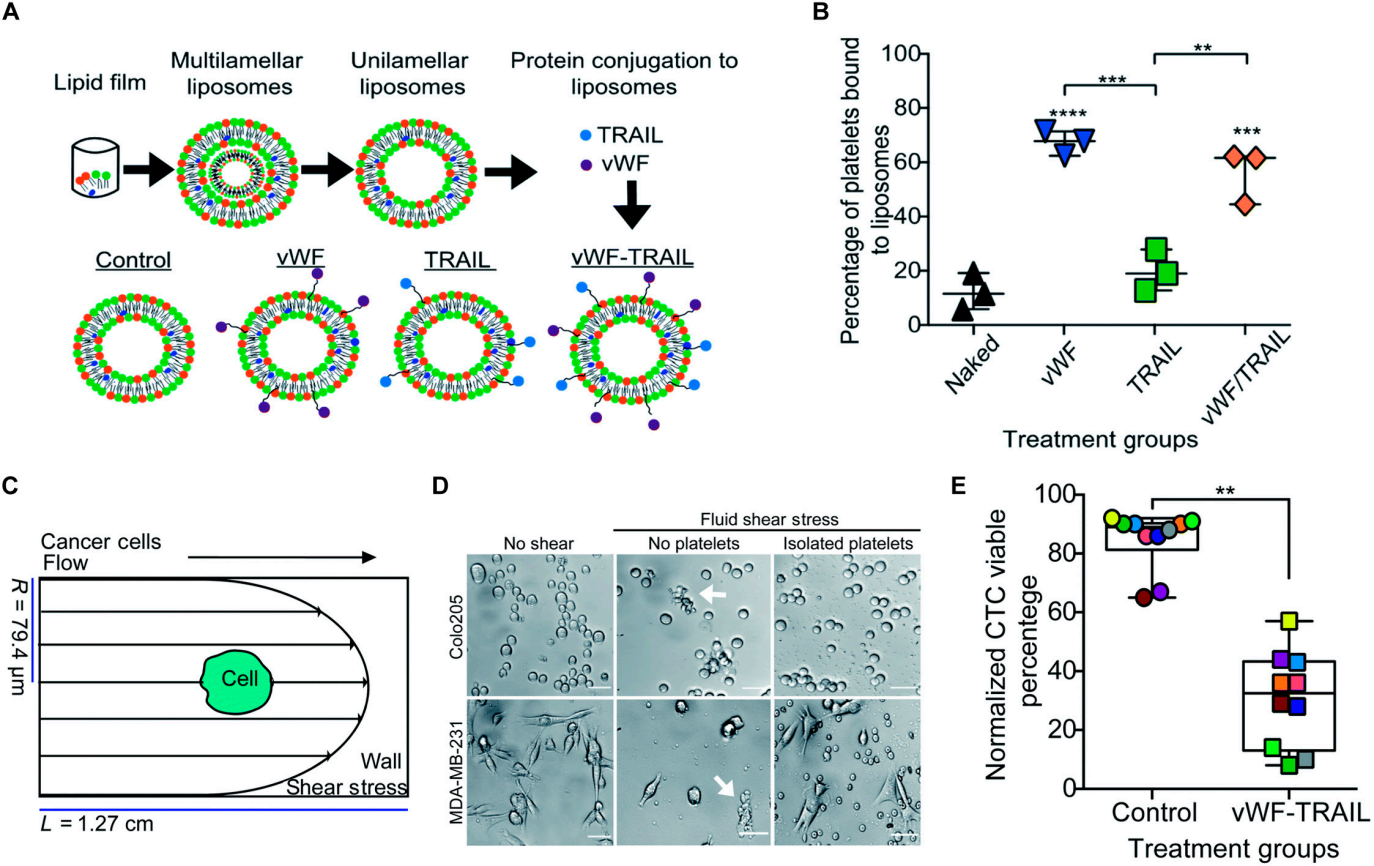
vWF-TRAIL on platelets effectively suppressed circulating tumor cells in the blood of cancer patients. (A) Fabrication process of TRAIL-decorated liposomes. (B) Percentage of platelets bound to liposomes. (C) Illustration of the fluid shear stress experienced by cancer cells. (D) Bright-field images of breast and colorectal cancer cells, exposing to fluid shear stress after incubation with or without of platelets. Scale bar: 40 μm. (E) Viability of circulating tumor cells after 4-h liposome treatment under physiological shear conditions (median ± range, *N* = 20 from 10 patients). (Figures were reproduced with copyright permission from Ref. [121].)

Most studies revealed platelets promoting cancer metastasis by attaching to the tumor circulating cell, thereby hindering immune cells. Therefore, Ortiz-Otero et al. developed TRAIL-decorated platelets by utilizing the binding of vWF (von Willebrand factor) A1 domain to the platelet receptor complex GPIb-IX-V, effectively inducing apoptosis in circulating colon cancer cells (COLO 205) and breast cancer (MDA-MB-231) cells. TRAIL-decorated platelets were produced as follows: (a) liposomes, featuring maleimide group, were decorated with thiolated proteins (TRAIL and vWF) via a maleimide-thiol reaction (Fig. 6A). (b) TRAIL-decorated liposome was subsequently interacted with vWFA1 domain on platelets (Fig. 6B) and (c) delivered TRAIL to cancer cells via the vWFA1 domain on platelets, and (d) surface-presented TRAIL killed CTCs. A key reason for using platelet cells is their ability to adhere to CTCs, forming a protective shield for CTCs from hemodynamic forces (5,920 dyn cm^−2^ for 1.08 ms) (Fig. 6C and D) and the cytotoxic effects of immune cells. To verify the cytotoxicity of these functional liposomes, blood samples from colorectal and breast cancer patients with metastatic tumors were collected and used. The results indicated that vWF-TRAIL liposomes eliminated 60% of the CTCs compared to control liposomes (Fig. 6E) [121]. This suggests that functionalized liposomes, combined with TRAIL and an adhering molecule, can be an effective tool for delivering TRAIL directly to CTCs, enhancing targeting and potentially suppressing secondary metastasis in patients. Table S3 summarizes various strategies of TRAIL decoration methods on vehicle surface.

Several living cell types serve as effective carriers for TRAIL delivery, with natural killer (NK) cells emerging as particularly promising due to their dual role as TRAIL carriers and immunotherapeutic agents [20]. While T cells are highly effective in targeting cancer cells, their aggressive immune responses can damage healthy tissues and trigger systemic immune overactivation, resulting in severe complications [122]. In contrast, NK cells provide a controlled immune response that reduces complications while ensuring effective treatment. Their intrinsic tumor-homing ability enhances precise TRAIL delivery, improving efficacy while minimizing off-target effects [123]. In this regard, TRAIL-conjugated liposomes were employed to NK cells for targeted delivery to oxaliplatin-resistant SW620 cells and metastatic COLO 205 colorectal cancer cells [20]. These TRAIL/anti-CD335 liposomes significantly enhanced NK cell-mediated apoptosis and demonstrated superior therapeutic efficacy under physiological shear stress in the lymphatics. Biodistribution analysis revealed prolonged presence of TRAIL liposomes in the spleen and tumor-draining mesenteric lymph nodes for at least 4 days, suggesting sustained therapeutic potential. These findings highlight NK cells as suitable candidates for TRAIL delivery, offering dual benefits of immune-mediated cytotoxicity and targeted TRAIL-induced apoptosis.

## Conclusion and Outlook

In this review, we have summarized recent advancements in TRAIL-based strategies that utilize various carriers, including NPs and gels. Although these TRAIL delivery systems are promising, several challenges should be addressed to further their clinical application. (a) The release kinetics of TRAIL need precise control to achieve an optimal rate that is neither too rapid nor too slow within the tumor region. This can be attained by developing finely tuned, programmable release profiles that utilize specific triggers, such as pH or enzymatic activity, to control TRAIL release using advanced engineered matrices. (b) The immunogenicity and potential toxicity of the carriers require careful consideration. Although PEGylated biomaterials are commonly used to enhance stability and prolong the half-life of therapeutic proteins, poly(ethylene glycol) (PEG) can induce immune reactions in some patients [124–126]. Therefore, there is a need to develop alternative materials that maintain the beneficial properties of PEG while reducing immunogenicity, which could enable the broader and safer use of biomaterial-based carriers. (c) The heterogeneity of target marker populations in cancer cells should be considered for improved cancer elimination. TRAIL-based therapies target TRAIL receptors, which are often overexpressed in tumor cells. However, not all cancers display the same level of TRAIL receptor expression, and insufficient expression can result in continued cancer cell survival and proliferation. To counter this, combining TRAIL-based therapies with other cancer targeting agents or cancer diagnostic markers may enhance therapeutic efficacy and improve cancer cell elimination [127–129]. In light of these considerations, we believe that the precise design of TRAIL delivery platforms holds significant promise for future research and clinical development.

## Data Availability

No data was used for the research described in the article.

## References

[1] Baskar R, Lee KA, Yeo R, Yeoh KW. Cancer and radiation therapy: Current advances and future directions. Int J Med Sci. 2012;9(3):193–199. 22408567 10.7150/ijms.3635 PMC3298009

[2] Anand U, Dey A, Chandel AKS, Sanyal R, Mishra A, Pandey DK, de Falco V, Upadhyay A, Kandimalla R, Chaudhary A, et al. Cancer chemotherapy and beyond: Current status, drug candidates, associated risks and progress in targeted therapeutics. Genes Dis. 2023;10(4):1367–1401. 37397557 10.1016/j.gendis.2022.02.007 PMC10310991

[3] Juthani R, Punatar S, Mittra I. New light on chemotherapy toxicity and its prevention. BJC Rep. 2024;2(1):41. 39516565 10.1038/s44276-024-00064-8 PMC11524128

[4] Vaddavalli PL, Schumacher B. The p53 network: Cellular and systemic DNA damage responses in cancer and aging. Trends Genet. 2022;38(6):598–612. 35346511 10.1016/j.tig.2022.02.010

[5] Zamai L, Ahmad M, Bennett IM, Azzoni L, Alnemri ES, Perussia B. Natural killer (NK) cell-mediated cytotoxicity: Differential use of TRAIL and Fas ligand by immature and mature primary human NK cells. J Exp Med. 1998;188(12):2375–2380. 9858524 10.1084/jem.188.12.2375 PMC2212426

[6] Myers JA, Miller JS. Exploring the NK cell platform for cancer immunotherapy. Nat Rev Clin Oncol. 2021;18(2):85–100. 32934330 10.1038/s41571-020-0426-7 PMC8316981

[7] Ogasawara J, Watanabe-Fukunaga R, Adachi M, Matsuzawa A, Kasugai T, Kitamura Y, Itoh N, Suda T, Nagata S. Lethal effect of the anti-Fas antibody in mice. Nature. 1993;364(6440):806–809. 7689176 10.1038/364806a0

[8] Wang S, El-Deiry WS. TRAIL and apoptosis induction by TNF-family death receptors. Oncogene. 2003;22(53):8628–8633. 14634624 10.1038/sj.onc.1207232

[9] Medema JP, Scaffidi C, Kischkel FC, Shevchenko A, Mann M, Krammer PH, Peter ME. FLICE is activated by association with the CD95 death-inducing signaling complex (DISC). EMBO J. 1997;16(10):2794–2804. 9184224 10.1093/emboj/16.10.2794 PMC1169888

[10] Johnstone RW, Frew AJ, Smyth MJ. The TRAIL apoptotic pathway in cancer onset, progression and therapy. Nat Rev Cancer. 2008;8(10):782–798. 18813321 10.1038/nrc2465

[11] Subbiah V, Brown RE, Buryanek J, Trent J, Ashkenazi A, Herbst R, Kurzrock R. Targeting the apoptotic pathway in chondrosarcoma using recombinant human Apo2L/TRAIL (dulanermin), a dual proapoptotic receptor (DR4/DR5) agonist. Mol Cancer Ther. 2012;11(11):2541–2546. 22914439 10.1158/1535-7163.MCT-12-0358 PMC3496030

[12] Herbst RS, Eckhardt SG, Kurzrock R, Ebbinghaus S, O’Dwyer PJ, Gordon MS, Novotny W, Goldwasser MA, Tohnya TM, Lum BL, et al. Phase I dose-escalation study of recombinant human Apo2L/TRAIL, a dual proapoptotic receptor agonist, in patients with advanced cancer. J Clin Oncol. 2010;28(17):2839–2846. 20458040 10.1200/JCO.2009.25.1991

[13] Lemke J, von Karstedt S, Zinngrebe J, Walczak H. Getting TRAIL back on track for cancer therapy. Cell Death Differ. 2014;21(9):1350–1364. 24948009 10.1038/cdd.2014.81 PMC4131183

[14] Lim SM, Kim TH, Jiang HH, Park CW, Lee S, Chen X, Lee KC. Improved biological half-life and anti-tumor activity of TNF-related apoptosis-inducing ligand (TRAIL) using PEG-exposed nanoparticles. Biomaterials. 2011;32(13):3538–3546. 21306770 10.1016/j.biomaterials.2011.01.054

[15] Thapa B, Kc R, Uludag H. TRAIL therapy and prospective developments for cancer treatment. J Control Release. 2020;326:335–349. 32682900 10.1016/j.jconrel.2020.07.013

[16] de Looff M, de Jong S, Kruyt FAE. Multiple interactions between cancer cells and the tumor microenvironment modulate TRAIL signaling: Implications for TRAIL receptor targeted therapy. Front Immunol. 2019;10:1530. 31333662 10.3389/fimmu.2019.01530 PMC6617985

[17] Liu H, Su D, Zhang J, Ge S, Li Y, Wang F, Gravel M, Roulston A, Song Q, Xu W, et al. Improvement of pharmacokinetic profile of TRAIL via trimer-tag enhances its antitumor activity in vivo. Sci Rep. 2017;7(1):8953. 28827692 10.1038/s41598-017-09518-1 PMC5566391

[18] da Silva WN, Carvalho Costa PA, Scalzo Junior SRA, Ferreira HAS, Prazeres P, Campos CLV, Rodrigues Alves MT, Da Silva NJA, De Castro Santos AL, Guimarães LC, et al. Ionizable lipid nanoparticle-mediated TRAIL mRNA delivery in the tumor microenvironment to inhibit colon cancer progression. Int J Nanomedicine. 2024;19:2655–2673. 38500680 10.2147/IJN.S452896 PMC10946446

[19] Guimaraes PPG, Gaglione S, Sewastianik T, Carrasco RD, Langer R, Mitchell MJ. Nanoparticles for immune cytokine TRAIL-based cancer therapy. ACS Nano. 2018;12(2):912–931. 29378114 10.1021/acsnano.7b05876 PMC5834400

[20] Greenlee JD, Zhang Z, Subramanian T, Liu K, King MR. TRAIL-conjugated liposomes that bind natural killer cells to induce colorectal cancer cell apoptosis. J Biomed Mater Res A. 2024;112(1):110–120. 37772330 10.1002/jbm.a.37621 PMC10794038

[21] Chen Z, Hu Q, Gu Z. Leveraging engineering of cells for drug delivery. Acc Chem Res. 2018;51(3):668–677. 29446615 10.1021/acs.accounts.7b00526

[22] Choi A, Javius-Jones K, Hong S, Park H. Cell-based drug delivery systems with innate homing capability as a novel nanocarrier platform. Int J Nanomedicine. 2023;18:509–525. 36742991 10.2147/IJN.S394389 PMC9893846

[23] Huang HC, Sung YC, Li CP, Wan D, Chao PH, Tseng YT, Liao BW, Cheng HT, Hsu FF, Huang CC, et al. Reversal of pancreatic desmoplasia by a tumour stroma-targeted nitric oxide nanogel overcomes TRAIL resistance in pancreatic tumours. Gut. 2022;71(9):1843–1855. 34921062 10.1136/gutjnl-2021-325180 PMC9380514

[24] Nguyen TTK, Woo SM, Seo SU, Banstola A, Kim H, Duwa R, Vu ATT, Hong IS, Kwon TK, Yook S. Enhanced anticancer efficacy of TRAIL-conjugated and odanacatib-loaded PLGA nanoparticles in TRAIL resistant cancer. Biomaterials. 2025;312: Article 122733. 39106819 10.1016/j.biomaterials.2024.122733

[25] Cao L, Du P, Jiang SH, Jin GH, Huang QL, Hua ZC. Enhancement of antitumor properties of TRAIL by targeted delivery to the tumor neovasculature. Mol Cancer Ther. 2008;7(4):851–861. 18413798 10.1158/1535-7163.MCT-07-0533

[26] Belyanskaya LL, Marti TM, Hopkins-Donaldson S, Kurtz S, Felley-Bosco E, Stahel RA. Human agonistic TRAIL receptor antibodies Mapatumumab and Lexatumumab induce apoptosis in malignant mesothelioma and act synergistically with cisplatin. Mol Cancer. 2007;6:66. 17953743 10.1186/1476-4598-6-66 PMC2134932

[27] Bellail AC, Qi L, Mulligan P, Chhabra V, Hao C. TRAIL agonists on clinical trials for cancer therapy: The promises and the challenges. Rev Recent Clin Trials. 2009;4(1):34–41. 19149761 10.2174/157488709787047530

[28] Forero-Torres A, Shah J, Wood T, Posey J, Carlisle R, Copigneaux C, Luo F(R, Wojtowicz-Praga S, Percent I, Saleh M. Phase I trial of weekly tigatuzumab, an agonistic humanized monoclonal antibody targeting death receptor 5 (DR5). Cancer Biother Radiopharm. 2010;25(1):13–19. 20187792 10.1089/cbr.2009.0673 PMC2883819

[29] Soria JC, Mark Z, Zatloukal P, Szima B, Albert I, Juhasz E, Pujol J-L, Kozielski J, Baker N, Smethurst D, et al. Randomized phase II study of dulanermin in combination with paclitaxel, carboplatin, and bevacizumab in advanced non-small-cell lung cancer. J Clin Oncol. 2011;29(33):4442–4451. 22010015 10.1200/JCO.2011.37.2623

[30] Geng C, Hou J, Zhao Y, Ke X, Wang Z, Qiu L, Xi H, Wang F, Wei N, Liu Y, et al. A multicenter, open-label phase II study of recombinant CPT (circularly permuted TRAIL) plus thalidomide in patients with relapsed and refractory multiple myeloma. Am J Hematol. 2014;89(11):1037–1042. 25092564 10.1002/ajh.23822

[31] Merchant MS, Geller JI, Baird K, Chou AJ, Galli S, Charles A, Amaoko M, Rhee EH, Price A, Wexler LH, et al. Phase I trial and pharmacokinetic study of lexatumumab in pediatric patients with solid tumors. J Clin Oncol. 2012;30(33):4141–4147. 23071222 10.1200/JCO.2012.44.1055 PMC3494837

[32] Paz-Ares L, Bálint B, de Boer RH, van Meerbeeck JP, Wierzbicki R, De Souza P, Galimi F, Haddad V, Sabin T, Hei Y-j, et al. A randomized phase 2 study of paclitaxel and carboplatin with or without conatumumab for first-line treatment of advanced non-small-cell lung cancer. J Thorac Oncol. 2013;8(3):329–337. 23370314 10.1097/JTO.0b013e31827ce554

[33] Reck M, Krzakowski M, Chmielowska E, Sebastian M, Hadler D, Fox T, Wang Q, Greenberg J, Beckman RA, von Pawel J. A randomized, double-blind, placebo-controlled phase 2 study of tigatuzumab (CS-1008) in combination with carboplatin/paclitaxel in patients with chemotherapy-naive metastatic/unresectable non-small cell lung cancer. Lung Cancer. 2013;82(3):441–448. 24148258 10.1016/j.lungcan.2013.09.014

[34] von Pawel J, Harvey JH, Spigel DR, Dediu M, Reck M, Cebotaru CL, Humphreys RC, Gribbin MJ, Fox NL, Camidge DR. Phase II trial of mapatumumab, a fully human agonist monoclonal antibody to tumor necrosis factor-related apoptosis-inducing ligand receptor 1 (TRAIL-R1), in combination with paclitaxel and carboplatin in patients with advanced non-small-cell lung cancer. Clin Lung Cancer. 2014;15(3):188–196.e2. 24560012 10.1016/j.cllc.2013.12.005

[35] Jain RK, Stylianopoulos T. Delivering nanomedicine to solid tumors. Nat Rev Clin Oncol. 2010;7(11):653–664. 20838415 10.1038/nrclinonc.2010.139 PMC3065247

[36] de Miguel D, Lemke J, Anel A, Walczak H, Martinez-Lostao L. Onto better TRAILs for cancer treatment. Cell Death Differ. 2016;23(5):733–747. 26943322 10.1038/cdd.2015.174 PMC4832109

[37] Kelley SK, Harris LA, Xie D, Deforge L, Totpal K, Bussiere J, Fox JA. Preclinical studies to predict the disposition of Apo2L/tumor necrosis factor-related apoptosis-inducing ligand in humans: Characterization of in vivo efficacy, pharmacokinetics, and safety. J Pharmacol Exp Ther. 2001;299(1):31–38. 11561060

[38] Montinaro A, Walczak H. Harnessing TRAIL-induced cell death for cancer therapy: A long walk with thrilling discoveries. Cell Death Differ. 2023;30(2):237–249. 36195672 10.1038/s41418-022-01059-z PMC9950482

[39] Martinez-Lostao L, García-Alvarez F, Basáñez G, Alegre-Aguarón E, Desportes P, Larrad L, Naval J, Martínez-Lorenzo MJ, Anel A. Liposome-bound APO2L/TRAIL is an effective treatment in a rabbit model of rheumatoid arthritis. Arthritis Rheum. 2010;62(8):2272–2282. 20506326 10.1002/art.27501

[40] Na SJ, Chae SY, Lee S, Park K, Kim K, Park JH, Kwon IC, Jeong SY, Lee KC. Stability and bioactivity of nanocomplex of TNF-related apoptosis-inducing ligand. Int J Pharm. 2008;363(1-2):149–154. 18694811 10.1016/j.ijpharm.2008.07.013

[41] Micheau O, Shirley S, Dufour F. Death receptors as targets in cancer. Br J Pharmacol. 2013;169(8):1723–1744. 23638798 10.1111/bph.12238 PMC3753832

[42] Xiao M, Tang Q, Zeng S, Yang Q, Yang X, Tong X, Zhu G, Lei L, Li S. Emerging biomaterials for tumor immunotherapy. Biomater Res. 2023;27(1):47. 37194085 10.1186/s40824-023-00369-8 PMC10189985

[43] Yun S, Kim S, Kim K. Cellular membrane components-mediated cancer immunotherapeutic platforms. Macromol Biosci. 2023;23(11): Article e2300159. 37319369 10.1002/mabi.202300159

[44] Sykes EA, Chen J, Zheng G, Chan WC. Investigating the impact of nanoparticle size on active and passive tumor targeting efficiency. ACS Nano. 2014;8(6):5696–5706. 24821383 10.1021/nn500299p

[45] Subhan MA, Yalamarty SSK, Filipczak N, Parveen F, Torchilin VP. Recent advances in tumor targeting via EPR effect for cancer treatment. J Pers Med. 2021;11(6):571. 34207137 10.3390/jpm11060571 PMC8234032

[46] Rosenblum D, Joshi N, Tao W, Karp JM, Peer D. Progress and challenges towards targeted delivery of cancer therapeutics. Nat Commun. 2018;9(1):1410. 29650952 10.1038/s41467-018-03705-y PMC5897557

[47] Karthikeyan C, Varaprasad K, Kim S, Kumar Jangid A, Lee W, Syedahamed Haja Hameed A, Kim K. Size-dependent cellular uptake of sodium alginate passivated tin dioxide nanoparticles in triple-negative breast cancer cells. J Ind Eng Chem. 2023;123:476–487.

[48] Amatya R, Hwang S, Park T, Min KA, Shin MC. In vitro and in vivo evaluation of PEGylated starch-coated iron oxide nanoparticles for enhanced photothermal cancer therapy. Pharmaceutics. 2021;13(6):871. 34204840 10.3390/pharmaceutics13060871 PMC8231641

[49] Liu D, Wu W, Ling J, Wen S, Gu N, Zhang X. Effective PEGylation of iron oxide nanoparticles for high performance in vivo cancer imaging. Adv Funct Mater. 2011;21(8):1498–1504.

[50] Mao W, Yoo HS. Inorganic nanoparticle functionalization strategies in immunotherapeutic applications. Biomater Res. 2024;28:0086. 39323561 10.34133/bmr.0086 PMC11423863

[51] Huang Y, Hsu JC, Koo H, Cormode DP. Repurposing ferumoxytol: Diagnostic and therapeutic applications of an FDA-approved nanoparticle. Theranostics. 2022;12(2):796–816. 34976214 10.7150/thno.67375 PMC8692919

[52] Sun C, Lee JSH, Zhang M. Magnetic nanoparticles in MR imaging and drug delivery. Adv Drug Deliv Rev. 2008;60(11):1252–1265. 18558452 10.1016/j.addr.2008.03.018 PMC2702670

[53] Veiseh O, Gunn JW, Zhang M. Design and fabrication of magnetic nanoparticles for targeted drug delivery and imaging. Adv Drug Deliv Rev. 2010;62(3):284–304. 19909778 10.1016/j.addr.2009.11.002 PMC2827645

[54] Sachdeva V, Monga A, Vashisht R, Singh D, Singh A, Bedi N. Iron oxide nanoparticles: The precise strategy for targeted delivery of genes, oligonucleotides and peptides in cancer therapy. J Drug Deliv Sci Technol. 2022;74: Article 103585.

[55] Muthiah M, Park IK, Cho CS. Surface modification of iron oxide nanoparticles by biocompatible polymers for tissue imaging and targeting. Biotechnol Adv. 2013;31(8):1224–1236. 23528431 10.1016/j.biotechadv.2013.03.005

[56] Perlstein B, Finniss SA, Miller C, Okhrimenko H, Kazimirsky G, Cazacu S, Lee HK, Lemke N, Brodie S, Umansky F, et al. TRAIL conjugated to nanoparticles exhibits increased anti-tumor activities in glioma cells and glioma stem cells in vitro and in vivo. Neuro-Oncology. 2013;15(1):29–40. 23144078 10.1093/neuonc/nos248 PMC3534416

[57] Shi Y, Wang J, Liu J, Lin G, Xie F, Pang X, Pei Y, Cheng Y, Zhang Y, Lin Z, et al. Oxidative stress-driven DR5 upregulation restores TRAIL/Apo2L sensitivity induced by iron oxide nanoparticles in colorectal cancer. Biomaterials. 2020;233: Article 119753. 31923762 10.1016/j.biomaterials.2019.119753

[58] Danhier F, Feron O, Preat V. To exploit the tumor microenvironment: Passive and active tumor targeting of nanocarriers for anti-cancer drug delivery. J Control Release. 2010;148(2):135–146. 20797419 10.1016/j.jconrel.2010.08.027

[59] Duan L, Yang F, He W, Song L, Qiu F, Xu N, Xu L, Zhang Y, Hua Z, Gu N. A multi-gradient targeting drug delivery system based on RGD-l-TRAIL-labeled magnetic microbubbles for cancer theranostics. Adv Funct Mater. 2016;26(45):8313–8324.

[60] Wang K, Kievit FM, Jeon M, Silber JR, Ellenbogen RG, Zhang M. Nanoparticle-mediated target delivery of TRAIL as gene therapy for glioblastoma. Adv Healthc Mater. 2015;4(17):2719–2726. 26498165 10.1002/adhm.201500563 PMC4715716

[61] Teraphongphom N, Chhour P, Eisenbrey JR, Naha PC, Witschey WR, Opasanont B, Jablonowski L, Cormode DP, Wheatley MA. Nanoparticle loaded polymeric microbubbles as contrast agents for multimodal imaging. Langmuir. 2015;31(43):11858–11867. 26446176 10.1021/acs.langmuir.5b03473 PMC4818153

[62] Duan L, Yang F, Song L, Fang K, Tian J, Liang Y, Li M, Xu N, Chen Z, Zhang Y, et al. Controlled assembly of magnetic nanoparticles on microbubbles for multimodal imaging. Soft Matter. 2015;11(27):5492–5500. 26061750 10.1039/c5sm00864f

[63] Veiseh O, Gunn JW, Kievit FM, Sun C, Fang C, Lee JS, Zhang M. Inhibition of tumor-cell invasion with chlorotoxin-bound superparamagnetic nanoparticles. Small. 2009;5(2):256–264. 19089837 10.1002/smll.200800646 PMC2692352

[64] Veiseh O, Sun C, Fang C, Bhattarai N, Gunn J, Kievit F, du K, Pullar B, Lee D, Ellenbogen RG, et al. Specific targeting of brain tumors with an optical/magnetic resonance imaging nanoprobe across the blood-brain barrier. Cancer Res. 2009;69(15):6200–6207. 19638572 10.1158/0008-5472.CAN-09-1157 PMC2742601

[65] Zaiki Y, Iskandar A, Wong TW. Functionalized chitosan for cancer nano drug delivery. Biotechnol Adv. 2023;67: Article 108200. 37331671 10.1016/j.biotechadv.2023.108200

[66] Stephen ZR, Kievit FM, Veiseh O, Chiarelli PA, Fang C, Wang K, Hatzinger SJ, Ellenbogen RG, Silber JR, Zhang M. Redox-responsive magnetic nanoparticle for targeted convection-enhanced delivery of O6-benzylguanine to brain tumors. ACS Nano. 2014;8(10):10383–10395. 25247850 10.1021/nn503735w PMC4212796

[67] Kumar CS, Mohammad F. Magnetic nanomaterials for hyperthermia-based therapy and controlled drug delivery. Adv Drug Deliv Rev. 2011;63(9):789–808. 21447363 10.1016/j.addr.2011.03.008 PMC3138885

[68] Revia RA, Zhang M. Magnetite nanoparticles for cancer diagnosis, treatment, and treatment monitoring: Recent advances. Mater Today. 2016;19(3):157–168. 10.1016/j.mattod.2015.08.022 PMC4981486 27524934

[69] Belkahla H, Mazarío E, Sangnier AP, Lomas JS, Gharbi T, Ammar S, Micheau O, Wilhelm C, Hémadi M. TRAIL acts synergistically with iron oxide nanocluster-mediated magneto- and photothermia. Theranostics. 2019;9(20):5924–5936. 31534529 10.7150/thno.36320 PMC6735372

[70] Deng X, Shao Z, Zhao Y. Solutions to the drawbacks of photothermal and photodynamic cancer therapy. Adv Sci. 2021;8(3):2002504. 10.1002/advs.202002504 PMC7856884 33552860

[71] Liu Y, Bhattarai P, Dai Z, Chen X. Photothermal therapy and photoacoustic imaging via nanotheranostics in fighting cancer. Chem Soc Rev. 2019;48(7):2053–2108. 30259015 10.1039/c8cs00618k PMC6437026

[72] Brin E, Wu K, Dagostino E, Meng-Chiang Kuo M, He Y, Shia WJ, Chen LC, Stempniak M, Hickey R, Almassy R, et al. TRAIL stabilization and cancer cell sensitization to its pro-apoptotic activity achieved through genetic fusion with arginine deiminase. Oncotarget. 2018;9(97):36914–36928. 30651925 10.18632/oncotarget.26398 PMC6319333

[73] Kim K, Chen WCW, Heo Y, Wang Y. Polycations and their biomedical applications. Prog Polym Sci. 2016;60:18–50.

[74] Chu H, Gao J, Wang Y. Design, synthesis, and biocompatibility of an arginine-based polyester. Biotechnol Prog. 2012;28(1):257–264. 22034156 10.1002/btpr.728

[75] Cho H, Kim J, Kim S, Jung YC, Wang Y, Kang BJ, Kim K. Dual delivery of stem cells and insulin-like growth factor-1 in coacervate-embedded composite hydrogels for enhanced cartilage regeneration in osteochondral defects. J Control Release. 2020;327:284–295. 32763434 10.1016/j.jconrel.2020.08.002

[76] Lee KW, Johnson NR, Gao J, Wang Y. Human progenitor cell recruitment via SDF-1alpha coacervate-laden PGS vascular grafts. Biomaterials. 2013;34(38):9877–9885. 24060423 10.1016/j.biomaterials.2013.08.082 PMC3882008

[77] Kim S, Lee J, Hwang MP, Wang Y, Kim K. Influence of fiber architecture and growth factor formulation on osteoblastic differentiation of mesenchymal stem cells in coacervate-coated electrospun fibrous scaffolds. J Ind Eng Chem. 2019;79:236–244.

[78] Lee MS, Ahmad T, Lee J, Awada HK, Wang Y, Kim K, Shin H, Yang HS. Dual delivery of growth factors with coacervate-coated poly(lactic-co-glycolic acid) nanofiber improves neovascularization in a mouse skin flap model. Biomaterials. 2017;124:65–77. 28188996 10.1016/j.biomaterials.2017.01.036

[79] Park U, Lee MS, Jeon J, Lee S, Hwang MP, Wang Y, Yang HS, Kim K. Coacervate-mediated exogenous growth factor delivery for scarless skin regeneration. Acta Biomater. 2019;90:179–191. 30936036 10.1016/j.actbio.2019.03.052

[80] Kim S, Jwa Y, Hong J, Kim K. Inhibition of colon cancer recurrence via exogenous TRAIL delivery using gel-like coacervate microdroplets. Gels. 2022;8(7):427. 35877512 10.3390/gels8070427 PMC9319433

[81] Caló E, Khutoryanskiy VV. Biomedical applications of hydrogels: A review of patents and commercial products. Eur Polym J. 2015;65:252–267.

[82] Vermonden T, Klumperman B. The past, present and future of hydrogels. Eur Polym J. 2015;72:341–343.

[83] Farasati Far B, Safaei M, Nahavandi R, Gholami A, Naimi-Jamal MR, Tamang S, Ahn JE, Ramezani Farani M, Huh YS. Hydrogel encapsulation techniques and its clinical applications in drug delivery and regenerative medicine: A systematic review. ACS Omega. 2024;9(27):29139–29158. 39005800 10.1021/acsomega.3c10102 PMC11238230

[84] Lee W, Shin MJ, Kim S, Lee CE, Choi J, Koo HJ, Choi MJ, Kim JH, Kim K. Injectable composite hydrogels embedded with gallium-based liquid metal particles for solid breast cancer treatment via chemo-photothermal combination. Acta Biomater. 2024;180:140–153. 38604467 10.1016/j.actbio.2024.04.011

[85] Lima-Sousa R, Alves CG, Melo BL, Costa FJP, Nave M, Moreira AF, Mendonça AG, Correia IJ, de Melo-Diogo D. Injectable hydrogels for the delivery of nanomaterials for cancer combinatorial photothermal therapy. Biomater Sci. 2023;11(18):6082–6108. 37539702 10.1039/d3bm00845b

[86] Li J, Mooney DJ. Designing hydrogels for controlled drug delivery. Nat Rev Mater. 2016;1(12):16071. 29657852 10.1038/natrevmats.2016.71 PMC5898614

[87] Jiang T, Shen S, Wang T, Li M, He B, Mo R. A substrate-selective enzyme-catalysis assembly strategy for oligopeptide hydrogel-assisted combinatorial protein delivery. Nano Lett. 2017;17(12):7447–7454. 29172544 10.1021/acs.nanolett.7b03371

[88] Rock KL, Reits E, Neefjes J. Present yourself! By MHC class I and MHC class II molecules. Trends Immunol. 2016;37(11):724–737. 27614798 10.1016/j.it.2016.08.010 PMC5159193

[89] Martín-Orozco N, Isibasi A, Ortiz-Navarrete V. Macrophages present exogenous antigens by class I major histocompatibility complex molecules via a secretory pathway as a consequence of interferon-gamma activation. Immunology. 2001;103(1):41–48. 11380691 10.1046/j.1365-2567.2001.01226.x PMC1783223

[90] Wu X, Li T, Jiang R, Yang X, Guo H, Yang R. Targeting MHC-I molecules for cancer: Function, mechanism, and therapeutic prospects. Mol Cancer. 2023;22(1):194. 38041084 10.1186/s12943-023-01899-4 PMC10693139

[91] Sykulev Y. Factors contributing to the potency of CD8^+^ T cells. Trends Immunol. 2023;44(9):693–700. 37558570 10.1016/j.it.2023.07.005 PMC10511257

[92] Colonna M. Natural killer cell receptors specific for MHC class I molecules. Curr Opin Immunol. 1996;8(1):101–107. 8729453 10.1016/s0952-7915(96)80112-9

[93] Dhatchinamoorthy K, Colbert JD, Rock KL. Cancer immune evasion through loss of MHC class I antigen presentation. Front Immunol. 2021;12: Article 636568. 33767702 10.3389/fimmu.2021.636568 PMC7986854

[94] Siegler EL, Kim YJ, Chen X, Siriwon N, Mac J, Rohrs JA, Bryson PD, Wang P. Combination cancer therapy using chimeric antigen receptor-engineered natural killer cells as drug carriers. Mol Ther. 2017;25(12):2607–2619. 28919377 10.1016/j.ymthe.2017.08.010 PMC5768663

[95] Im S, Jang D, Saravanakumar G, Lee J, Kang Y, Lee YM, Lee J, Doh J, Yang ZY, Jang MH, et al. Harnessing the formation of natural killer-tumor cell immunological synapses for enhanced therapeutic effect in solid tumors. Adv Mater. 2020;32(22): Article e2000020. 32319126 10.1002/adma.202000020

[96] Fares J, Fares MY, Khachfe HH, Salhab HA, Fares Y. Molecular principles of metastasis: A hallmark of cancer revisited. Signal Transduct Target Ther. 2020;5(1):28. 32296047 10.1038/s41392-020-0134-x PMC7067809

[97] Luzzi KJ, MacDonald IC, Schmidt EE, Kerkvliet N, Morris VL, Chambers AF, Groom AC. Multistep nature of metastatic inefficiency: Dormancy of solitary cells after successful extravasation and limited survival of early micrometastases. Am J Pathol. 1998;153(3):865–873. 9736035 10.1016/S0002-9440(10)65628-3 PMC1853000

[98] Chambers AF, Groom AC, MacDonald IC. Dissemination and growth of cancer cells in metastatic sites. Nat Rev Cancer. 2002;2(8):563–572. 12154349 10.1038/nrc865

[99] Gao Y, Yuan Z. Nanotechnology for the detection and kill of circulating tumor cells. Nanoscale Res Lett. 2014;9(1):500. 25258614 10.1186/1556-276X-9-500 PMC4174536

[100] Zhang Z, King MR. Nanomaterials for the capture and therapeutic targeting of circulating tumor cells. Cell Mol Bioeng. 2017;10(4):275–294. 28804522 10.1007/s12195-017-0497-4 PMC5533815

[101] Steeg PS. Tumor metastasis: Mechanistic insights and clinical challenges. Nat Med. 2006;12(8):895–904. 16892035 10.1038/nm1469

[102] Anderson NR, Minutolo NG, Gill S, Klichinsky M. Macrophage-based approaches for cancer immunotherapy. Cancer Res. 2021;81(5):1201–1208. 33203697 10.1158/0008-5472.CAN-20-2990

[103] Luginbuehl V, Abraham E, Kovar K, Flaaten R, Muller AMS. Better by design: What to expect from novel CAR-engineered cell therapies? Biotechnol Adv. 2022;58: Article 107917. 35149146 10.1016/j.biotechadv.2022.107917

[104] Zhu Q, Huang X, Deng B, Guan L, Zhou H, Shi B, Liu J, Shan X, Fang X, Xu F, et al. Tumor micro-environment induced TRAIL secretion from engineered macrophages for anti-tumor therapy. Cell Immunol. 2024;403-404: Article 104857. 39032210 10.1016/j.cellimm.2024.104857

[105] Ryan JM, Barry FP, Murphy JM, Mahon BP. Mesenchymal stem cells avoid allogeneic rejection. J Inflamm. 2005;2:8. 10.1186/1476-9255-2-8 PMC1215510 16045800

[106] Wu X, Jiang J, Gu Z, Zhang J, Chen Y, Liu X. Mesenchymal stromal cell therapies: Immunomodulatory properties and clinical progress. Stem Cell Res Ther. 2020;11(1):345. 32771052 10.1186/s13287-020-01855-9 PMC7414268

[107] Spaeth E, Klopp A, Dembinski J, Andreeff M, Marini F. Inflammation and tumor microenvironments: Defining the migratory itinerary of mesenchymal stem cells. Gene Ther. 2008;15(10):730–738. 18401438 10.1038/gt.2008.39

[108] TomyTomcy A, Sindhu ER. Mesenchymal stem cells—An excellent therapeutic agent for cancer. Asia Pac J Clin Oncol. 2024;20(1):7–15. 37190944 10.1111/ajco.13969

[109] Tang XJ, Lu JT, Tu HJ, Huang KM, Fu R, Cao G, Huang M, Cheng LH, Dai LJ, Zhang L. TRAIL-engineered bone marrow-derived mesenchymal stem cells: TRAIL expression and cytotoxic effects on C6 glioma cells. Anticancer Res. 2014;34(2):729–734. 24511006

[110] Lee CE, Kim S, Park HW, Lee W, Jangid AK, Choi Y, Jeong WJ, Kim K. Tailoring tumor-recognizable hyaluronic acid-lipid conjugates to enhance anticancer efficacies of surface-engineered natural killer cells. Nano Converg. 2023;10(1):56. 38097911 10.1186/s40580-023-00406-1 PMC10721593

[111] Kim S, Li S, Jangid AK, Park HW, Lee DJ, Jung HS, Kim K. Surface engineering of natural killer cells with CD44-targeting ligands for augmented cancer immunotherapy. Small. 2024;20(24): Article e2306738. 38161257 10.1002/smll.202306738

[112] Layek B, Sadhukha T, Prabha S. Glycoengineered mesenchymal stem cells as an enabling platform for two-step targeting of solid tumors. Biomaterials. 2016;88:97–109. 26946263 10.1016/j.biomaterials.2016.02.024

[113] Cheng H, Byrska-Bishop M, Zhang CT, Kastrup CJ, Hwang NS, Tai AK, Lee WW, Xu X, Nahrendorf M, Langer R, et al. Stem cell membrane engineering for cell rolling using peptide conjugation and tuning of cell-selectin interaction kinetics. Biomaterials. 2012;33(20):5004–5012. 22494889 10.1016/j.biomaterials.2012.03.065 PMC3366278

[114] Noh KM, Jangid AK, Park J, Kim S, Kim K. Membrane-immobilized gemcitabine for cancer-targetable NK cell surface engineering. J Mater Chem B. 2024;12(46):12087–12102. 39465499 10.1039/d4tb01639d

[115] Kim S, Lee DH, Park HW, Noh KM, Jangid AK, Park H, Kim GJ, Kim K. Amphiphilic lipid conjugate-mediated surface engineering of placenta-derived mesenchymal stem cells for alleviating liver damage and fibrosis. Chem Eng J. 2025;503: Article 158313.

[116] Kim S, Li S, Gajendiran M, Jangid AK, Lee D-J, Jung H-S, Kim K. Lipid anchor-mediated NK cell surface engineering for enhanced cancer immunotherapy. Chem Eng J. 2023;473: Article 145211.

[117] Wu X, Wang S, Li M, Wang A, Zhou Y, Li P, Wang Y. Nanocarriers for TRAIL delivery: Driving TRAIL back on track for cancer therapy. Nanoscale. 2017;9(37):13879–13904. 28914952 10.1039/c7nr04959e

[118] Yun H, Su W, You T, Wang J, Ying Y, Wang C, Ren Y, Lu B, Li Y, Liu C. Boosting physical performance in SD rats through brain-targeted delivery of caffeine-loaded transferrin liposomes. Heliyon. 2024;10(14): Article e34617. 39114047 10.1016/j.heliyon.2024.e34617 PMC11305279

[119] Mitchell MJ, Wayne E, Rana K, Schaffer CB, King MR. TRAIL-coated leukocytes that kill cancer cells in the circulation. Proc Natl Acad Sci USA. 2014;111(3):930–935. 24395803 10.1073/pnas.1316312111 PMC3903223

[120] Jyotsana N, Zhang Z, Himmel LE, Yu F, King MR. Minimal dosing of leukocyte targeting TRAIL decreases triple-negative breast cancer metastasis following tumor resection. Sci Adv. 2019;5(7):eaaw4197. 31355333 10.1126/sciadv.aaw4197 PMC6656540

[121] Ortiz-Otero N, Marshall JR, Lash BW, King MR. Platelet mediated TRAIL delivery for efficiently targeting circulating tumor cells. Nanoscale Adv. 2020;2(9):3942–3953. 36132797 10.1039/d0na00271b PMC9419179

[122] Chen T, Wang M, Chen Y, Liu Y. Current challenges and therapeutic advances of CAR-T cell therapy for solid tumors. Cancer Cell Int. 2024;24(1):133. 38622705 10.1186/s12935-024-03315-3 PMC11017638

[123] Laskowski TJ, Biederstadt A, Rezvani K. Natural killer cells in antitumour adoptive cell immunotherapy. Nat Rev Cancer. 2022;22(10):557–575. 35879429 10.1038/s41568-022-00491-0 PMC9309992

[124] Chen BM, Cheng TL, Roffler SR. Polyethylene glycol immunogenicity: Theoretical, clinical, and practical aspects of anti-polyethylene glycol antibodies. ACS Nano. 2021;15(9):14022–14048. 34469112 10.1021/acsnano.1c05922

[125] Suk JS, Xu Q, Kim N, Hanes J, Ensign LM. PEGylation as a strategy for improving nanoparticle-based drug and gene delivery. Adv Drug Deliv Rev. 2016;99(Pt A):28–51. 26456916 10.1016/j.addr.2015.09.012 PMC4798869

[126] Shi L, Zhang J, Zhao M, Tang S, Cheng X, Zhang W, Li W, Liu X, Peng H, Wang Q. Effects of polyethylene glycol on the surface of nanoparticles for targeted drug delivery. Nanoscale. 2021;13(24):10748–10764. 34132312 10.1039/d1nr02065j

[127] Thi HN, Minh TV, Van DV, La Thi H, Le Thi HP, Nguyen VT, Dang LH, Tran NQ, Le Thi P. Stearyl polyoxyethylene-grafted heparin nanogel for oral delivery of cisplatin: Enhanced drug loading capacity and anticancer efficacy. Macromol Res. 2025;33:289–302.

[128] Janakiraman K, Jayaraj G, Sethuraman V, Krishnaswami V. Nano-engineered monoclonal antibodies expanding the newer avenues for cancer targeting. Macromol Res. 2024;33:117–135.

[129] Nguyen DT, Dang LH, Le HK, Ngan LT, Tran NQ, Park KD, Le Thi P. Injectable hyaluronic acid–cyclodextrin-based hydrogels for localized and sustained release of anticancer drugs. Macromol Res. 2024;32(8):777–788.

