# Supplementary material for: Recent Tumor Necrosis Factor-Related Apoptosis-Inducing Ligand Engineering Strategies for Precise Strike Therapy against Tumor
Source: Biomater Res. 2025 Mar 19;29:0170. doi: 10.34133/bmr.0170 (PMC11922527; doi:10.34133/bmr.0170)
Supplement: Supplementary 1 — Tables S1 to S3 [file bmr.0170.f1.doc]

**Front Matter**

Full title:

Recent Tumor necrosis factor-related apoptosis-inducing ligand (TRAIL) Engineering Strategies for Precise Strike Therapy Against Tumor

Short title:

TRAIL Engineering for Tumor Suppression

**Authors**

Chae Eun Lee^1†^, Kyung Mu Noh^1†^, Sungjun Kim^1^, Jiyeon Hong^2^, Kyobum Kim^1*^

**Affiliations**

^1^ Department of Chemical & Biochemical Engineering, Dongguk University, Seoul 04620, Republic of Korea

^2^ School of Chemical and Biological Engineering, Institute of Chemical Processes, Seoul National University, Seoul 08826, Republic of Korea

^†^ These authors contributed equally to this work

^*^Address correspondence to: kyobum.kim@dongguk.edu

**Supplementary Tables**

**Table S1.** rhTRAIL and TRAIL agonistic antibodies in clinical trials

| **Types** | **Name** | **Combination therapies** | **Mechanism** | **Cancer type** | **Dose (mg/kg)** | **Treatment schedule** | **Clinical Efficacy** | **Clinical trials** | **Ref.** |
| --- | --- | --- | --- | --- | --- | --- | --- | --- | --- |
| rhTRAIL | Dulanermin | Paclitaxel  Carboplatin  Bevacizumab | DR4 and DR5 activation | Advanced NSCLC | 8 or 20 | Once daily for 5 or 2 days | Safe  PR: 40, 40%  ORR: 40, 40% | Phase II | [29] |
| rhTRAIL | CPT | thalidomide | DR4 and DR5 activation | Multiple myeloma | 5, 8 or 10 | Once daily for 5 days | Safe  CR: 4.9%  PR: 9.8%  ORR: 22% | Phase II | [30] |
| TRAIL-R antibodies | Mapatumumab | Paclitaxel  Carboplatin | DR4 activation | Advanced NSCLC | 10 or 30 | Once every 3 weeks for an average of 8 and 6 cycles | Safe  CR: 0, 2.8%  PR: 13.5, 33.3%  ORR: 13.5, 36.1% | Phase II | [34] |
| TRAIL-R antibodies | Lexatumumab | None | DR5 activation | Solid tumors | 3, 5, 8, or 10 | Once daily for 14 days | Safe  CR: 0%  PR: 0% | Phase I | [31] |
| TRAIL-R antibodies | Conatumumab | Paclitaxel  Carboplatin | DR5 activation | Advanced NSCLC | 3 or 15 | Once every 3 weeks for a maximum 6 cycles | Safe  CR: 2, 0%  PR: 25, 27%  ORR: 27, 27% | Phase II | [32] |
| TRAIL-R antibodies | Tigatuzumab | Paclitaxel  Carboplatin | DR5 activation | Advanced NSCLC | 10 followed by 8 | Once every 3 weeks for a maximum 6 cycles | Safe  CR: 0%  PR: 24.5%  ORR: 24.5% | Phase II | [33] |

NSCLC, non-small cell lung cancer; CR, complete response; PR, partial response; ORR, overall response rate

**Table S2.** Biomaterial-based TRAIL delivery

| **TRAIL**  **delivery platform** | **Fabrication method** | **Tumor model** | **Mechanism** | **Therapeutic effects** | **Ref.** |
| --- | --- | --- | --- | --- | --- |
| Conjugation with iron oxide NPs | • Step 1: Coating the SPIONs with PEI_2K_  • Step 2: Functionalizing the PEI_2k_-coated SPIONs with TRAIL protein via physical interactions | Colorectal cancer (COLO-205) tumor-bearing mice | • ROS-mediated DR5 upregulation induced by iron oxide NPs | • About 64% of tumor suppression after 26 days  - Bolus TRAIL: about 15% | [57] |
| Conjugation with iron oxide NPs | • Step 1: Modification of PVA with carboxyl groups to enhance biocompatibility of MBs  • Step 2: Chemical attachment of APTS-modified SPIONs to the carboxylated MBs, forming MMBs  • Step 3: Conjugation of RGD-_L_-TRAIL proteins to the MMBs through reactions between the amino groups on the MMBs and the carboxyl groups of the proteins | Colon cancer (HT29) tumor-bearing mice | • Tumor treatment monitoring facilitated by MMBs through US and MRI  • Improved targeting efficiency of MMBs toward cancer cells by the RGD moiety | • About 46% of tumor suppression after 20 days  - Bolus RGD-_L_-TRAIL: about 50% | [59] |
| Conjugation with iron oxide NPs | • Step 1: Coating of iron oxide NPs with CP-PEG and complexation with TRAIL-encoding plasmid DNA  • Step 2: Conjugation of CTX to the NP-DNA complexes via a maleimide-thiol reaction using SIA and Traut’t reagent | Glioblastoma (T98G) tumor bearing mice | • Enhanced targeting efficiency toward glioblastoma by CTX  • Facilitation of tumor cell transfection and soluble TRAIL release by TRAIL-encoding DNA delivery | • About 78% of tumor suppression after 38 days | [60] |
| Encapsulation into mPEG-Coa | • Coulombic interactions between mPEG-poly (ethylene arginylaspartate diglyceride) and heparin | *In vitro* colon cancer (HCT-116) | • TRAIL release from mPEG-Coa under TME mimic condition (PBS with 185 mM NaCl) | • About 150% inhibition after 7 days  - Bolus TRAIL: about 50% | [80] |
| Loaded into hydrogel | • Self-assembly with hydrogel precursors (Fmoc-F and FF-Dopa, and W-NC) to form Fmoc-FFF-Dopa | Breast cancer (MDA-MB-231) tumor-bearing mice | • Local delivery of hydrogel, co-loaded with hirudin (antiangiogenic protein) and TRAIL | • Tumor removal within  5 days  - TRAIL-gel: tumors persisted for 9 days | [87] |
| Loaded into nanogel | • Step 1: Core of nanogel fabrication through a water-oil single emulsion using SF and phospholipid DOPA  • Step 2: Coating the SF nanogel with lipids, containing DSPE-PEG_2k_-maleimide  • Step 3: LQT28 peptide conjugation via maleimide-thiol reaction between the DSPE-PEG_2k_-maleimide and thiol group of peptide | Pancreatic ductal adenocarcinoma model | • Systematically delivery of hydrogel, conjugated with LQT28 peptide for cancer targeting and co-loaded with nitric oxide (anticancer agent) and TRAIL | • Reduced the tumor volume after 16 days of treatment to ~ 50 mm^3^  - Control: ~ 200 mm^3^ | [23] |

SPION, superparamagnetic iron oxide nanoparticle; PEI, polyethyleneimine; ROS, reactive oxidative stress; DR, death receptor; NP, nanoparticle; PVA, polyvinyl alcohol; MB, microbubble; APTS, aminopropyltriethoxysilane; MMB, magnetic microbubble; RGD, arginine-glycine-aspartic acid; US, ultrasound; MRI, magnetic resonance imaging; CP, chitosan-PEG; CTX, chlorotoxin; SIA, N-succinimidyl iodoacetate; mPEG-Coa, methoxy-poly (ethylene glycol) conjugated coacervates; TME, tumor microenvironment; Fmoc-F, Fmoc-Phe; FF-Dopa, Phe-Phe-Dopa; W-NC, WQ9-2 encapsulated within nanocapsule; SF, silk fibroin; DOPA, dihydroxyphenylalanine; DSPE-PEG_2k_-maleimide, 1,2-distearoyl-sn-glycero-3-phosphoethanolamine-N-[maleimide(polyethylene glycol)-2000]

**Table S3.** TRAIL decoration methods on vehicle surface

| **TRAIL-functionalized cell** | **Decoration method** | **Mechanism** | **Tumor model** | **Therapeutic outcome** | **Ref.** |
| --- | --- | --- | --- | --- | --- |
| Tri-TRAIL-iM | TRAIL expression on macrophage surface by gene transfection | • Step 1: High Arg1 expression in TME  • Step 2: Activation of Arg1 promotor of pCDH-Arg1-Tri-TRAIL in genetically modified macrophage  • Step 3: TRAIL secretion from genetically modified macrophages | Breast cancer tumor bearing mice (4T1): I.V. injection | • Inhibition of tumor volume with Tri-TRAIL-iM treatment (about 1400 mm^2^) after 23 days (PBS control: about 1800 mm^2^) | [104] |
| TRAIL-transfected MSCs | TRAIL expression on MSC surface by gene transfection | • Step 1: Migration of TRAIL expressed MSCs to cancer cell  • Step 2: Binding with DR5 receptor | *In vitro* glioblastoma cell (C6) | • Suppressed C6 cell growth to 16.7% after one day of co-culture (control plasmid: 63.7%) | [109] |
| ES/TRAIL liposomes | Binding interaction between Ni-NTA on liposome and his-tagged functional protein (TRAIL & ES) | • Step 1: Adhering with leukocyte membranes via ES binding of ES/TRAIL liposomes  • Step 2: Targeting TRAIL receptor on cancer cells | *In vivo* circulating colon cancer (COLO 205): I.V. injection | • Reduced number of viable cancer cells (<2,000 cells/mL) compared to control ES liposomes (~130,000 cells/mL) | [119] |
| ETL | Binding interaction between Ni-NTA on liposome and his-tagged functional protein (TRAIL & ES) | • Step 1: Adhering with leukocyte membranes via ES binding of ETL  • Step 2: Targeting TRAIL receptor on cancer cells | *In vivo* circulating breast cancer (4T1): I.V. injection | • A twofold reduction in 4T1 cells in ETL versus controls | [120] |
| TRAIL-  decorated platelet | Thiol-Maleimide click chemistry of maleimide on liposome and thiolated functional proteins (TRAIL and vWFA1 domain) | • Step 1: Liposome decoration on the platelet surface via vWFA1 binding to the platelet receptor  • Step 2: Targeted TRAIL delivery to cancer cells through the vWFA1 domain on platelets | Blood samples from colorectal and breast cancer patients | • Killed 60% of CTCs compared to control liposomes | [121] |

Tri-TRAIL-iM, trimeric TRAIL; TME, tumor microenvironment; MSCs, mesenchymal stem cells; DR, death receptor; ES/TRAIL liposomes, E-selectin and TRAIL-decorated liposomes; ES, E-selectin; Ni-NTA, nickel nitrilotriacetic acid; ETL, ES and TRAIL-functionalized liposome; vWF, von Willebrand Factor, CTCs, circulating tumor cell
